# Supplementary material for: Pharmacogenomics polygenic risk score for drug response prediction using PRS-PGx methods
Source: Nat Commun. 2022 Sep 8;13:5278. doi: 10.1038/s41467-022-32407-9 (PMC9458667; doi:10.1038/s41467-022-32407-9)
Supplement: Supplementary file 1 — Supplementary Information [file 41467_2022_32407_MOESM1_ESM.pdf]

Supplementary Information for "Pharmacogenomics Polygenic Risk Score for Drug Response Prediction Using PRS-PGx Methods"

Song Zhai[1,†], Hong Zhang[1,†], Devan V. Mehrotra[2] and Judong Shen[1,∗]

[1] Biostatistics and Research Decision Sciences, Merck & Co., Inc., Rahway, New Jersey, 07065, USA

[2] Biostatistics and Research Decision Sciences, Merck & Co., Inc., North Wales, Pennsylvania, 19454, USA

† These authors contributed equally

∗ Corresponding author: Judong Shen (judong.shen@merck.com)

Supplementary Methods

A. Proof of Equation (8)

$$\text{cor}^2(S_{PGx}, Y|T=1) = \frac{\text{cov}^2\left(\sum_{i=1}^m(\beta_i+\alpha_i)G_i,\ \sum_{i=1}^m(\beta_i+\alpha_i)G_i+\epsilon\right)}{\text{var}(\sum_{i=1}^m(\beta_i+\alpha_i)G_i)(\text{var}(\sum_{i=1}^m(\beta_i+\alpha_i)G_i)+\sigma^2)}$$

$$= \frac{(\text{var}(\sum_{i=1}^m(\beta_i+\alpha_i)G_i))^2}{\text{var}(\sum_{i=1}^m(\beta_i+\alpha_i)G_i)(\text{var}(\sum_{i=1}^m(\beta_i+\alpha_i)G_i)+\sigma^2)}$$

$$= \frac{\text{var}(\sum_{i=1}^m(\beta_i+\alpha_i)G_i)}{\text{var}(\sum_{i=1}^m(\beta_i+\alpha_i)G_i)+\sigma^2}.$$

B. Proof of Equation (9)

$$\begin{aligned}
\mathrm{cor}^2(S_{Dis}, Y|T=1) &= h^2 \cdot \frac{\mathrm{var}(\sum_{i=1}^m \beta_i \mathbf{G}_i) + \mathrm{cov}(\sum_{i=1}^m \beta_i \mathbf{G}_i,\ \sum_{i=1}^m \alpha_i \mathbf{G}_i)}{\mathrm{var}(\sum_{i=1}^m \beta_i \mathbf{G}_i)\,\mathrm{var}(\sum_{i=1}^m (\beta_i + \alpha_i)\mathbf{G}_i)} \\
&= h^2 \cdot \frac{(\sum_{i=1} \psi_i + \sum_{i=1} \rho_i \sqrt{\psi_i \xi_i})^2}{\sum_{i=1} \psi_i \sum_{i=1} (\psi_i + \xi_i + 2\rho_i \sqrt{\psi_i \xi_i})} \\
&= h^2 \cdot \frac{(\sum_{i=1} \psi_i)^2 + (\sum_{i=1} \rho_i \sqrt{\psi_i \xi_i})^2 + 2\sum_{i=1} \psi_i \sum_{i=1} \rho_i \sqrt{\psi_i \xi_i}}{(\sum_{i=1} \psi_i)^2 + \sum_{i=1} \psi_i \sum_{i=1} \xi_i + 2\sum_{i=1} \psi_i \sum_{i=1} \rho_i \sqrt{\psi_i \xi_i}} \\
&= h^2 \left(1 - \frac{\sum_{i=1} \psi_i \sum_{i=1} \xi_i - (\sum_{i=1}^m \rho_i \sqrt{\psi_i \xi_i})^2}{(\sum_{i=1}^m \psi_i)^2 + 2\sum_{i=1}^m \psi_i \sum_{i=1}^m \rho_i \sqrt{\psi_i \xi_i} + \sum_{i=1} \psi_i \sum_{i=1} \xi_i}\right).
\end{aligned}$$

The above deduction is under the fixed $\boldsymbol{\beta} = (\beta_1, ..., \beta_m)'$ and $\boldsymbol{\alpha} = (\alpha_1, ..., \alpha_m)'$.

Assume $\Sigma = (\sigma_{ij})$, β_j, α_j, $j = 1, ..., m$, are constants, equation (8) still holds because its proof does not rely on any properties of $\boldsymbol{\beta}$ and $\boldsymbol{\alpha}$. To derive equation similar to equation (9), we write

$$\begin{aligned}
\mathrm{cor}^2(S_{Dis}, Y|T=1) &= h^2 \cdot \frac{(\beta'\Sigma(\beta+\alpha))^2}{\beta'\Sigma\beta \cdot (\beta+\alpha)'\Sigma(\beta+\alpha)} \\
&= h^2 \cdot \frac{(\beta'\Sigma\beta)^2 + (\beta'\Sigma\alpha)^2 + 2(\beta'\Sigma\beta)(\beta'\Sigma\alpha)}{(\beta'\Sigma\beta)^2 + (\beta'\Sigma\beta)(\alpha'\Sigma\alpha) + 2(\beta'\Sigma\beta)(\beta'\Sigma\alpha)} \\
&= h^2 \left(1 - \frac{(\beta'\Sigma\beta)(\alpha'\Sigma\alpha) - (\beta'\Sigma\alpha)^2}{(\beta'\Sigma\beta)^2 + (\beta'\Sigma\beta)(\alpha'\Sigma\alpha) + 2(\beta'\Sigma\beta)(\beta'\Sigma\alpha)}\right).
\end{aligned}$$

By Cauchy-Schwarz inequality, $(\boldsymbol{\beta}'\Sigma\boldsymbol{\alpha})^2 \le (\boldsymbol{\beta}'\Sigma\boldsymbol{\beta})(\boldsymbol{\alpha}'\Sigma\boldsymbol{\alpha})$. Thus, $\mathrm{cor}^2(S_{Dis}, Y|T=1) \le h^2$. The equality holds if and only if $\boldsymbol{\beta}$ and $\boldsymbol{\alpha}$ are linearly dependent.

C. PRS-Dis-LDpred2

We directly use the disease PRS LDpred2 method[1] by LD blocks as the PRS-Dis-LDpred2 method (based on bigsnpr R package v1.8.1 https://cran.r-project.org/web/packages/bigsnpr/index.html). The previous version of LDpred2 is LDpred[2], which is a method that infers the posterior mean effect size of each genetic marker from disease GWAS summary statistics while accounting for LD, using a point-normal prior on the SNP effect sizes and LD information from an external reference panel. Consider the linear model

$$\mathbf{Y}_{n\times 1} = \mathbf{G}_{n\times m}\boldsymbol{\beta}_{m\times 1} + \boldsymbol{\epsilon}_{n\times 1},$$

where both the phenotype $\mathbf{Y}$ and the genotype matrix $\mathbf{G}$ have been standardized. LDpred replaces an independent point-normal prior on each regression coefficient β_j:

$$\beta_j \sim \begin{cases} \mathrm{N}(0, \dfrac{h^2}{mp}), & \text{with probability } p, \\ \quad 0, & \text{with probability } 1-p, \end{cases}$$

where h^2 is the heritability explained by genome-wide genetic markers, and p is the fraction of causal variants. In LDpred, p and h^2 are considered as hyper-parameters, and determined via grid searching.

As an updated version of LDpred, LDpred2-grid (Privé et al., 2020) introduces a third hyper-parameter indicating whether sparsity is enabled or not. When implementing the PRS-Dis-LDpred2 method, we tested a grid of hyper-parameters with p and h^2. We also enabled the sparsity by setting "sparse = TRUE", aimed at providing sparse effect size estimates, i.e., shrinking some effects to exactly 0.

Besides LDpred2-grid, Privé et al. (2020) also proposed another version called LDpred2-auto, which estimates p and h^2 within the model. This makes LDpred2-auto a method free of hyper-parameters. More specifically, to estimate p in the Gibbs sampler, LDpred2 counts the number of non-zero variants as $M_c = \sum_j (\beta_j \neq 0) \sim \text{Binomial}(m, p)$; and estimates

$h^2 = \beta^T \mathbf{R} \beta$, where $\mathbf{R}$ is the correlation matrix. To determine which method, LDpred2-grid or LDpred2-auto, should be used as PRS-Dis-LDpred2 function, we further conducted simulation studies following the same simulation method described in the main text. The heritability was fixed at 0.3 and $\psi/\xi = 1$. Numbers of the causal variants for P(causal) = 0.001, 0.01 and 0.1 were 5, 50 and 500, respectively. The training sample size was 20000. The tuning parameters of LDpred2-grid were selected via cross-validation in the training data. Supplementary Figure 13 indicated that although overall the two methods' performance was very close to each other, LDpred2-grid actually slightly outperformed LDpred2-auto in most scenarios (i.e., when the proportion of causal variants was small to moderate). Therefore in our study, we only used LDpred2-grid as the best disease PRS method to be compared with our proposed PRS-PGx approaches.

D. Obtain D from LD Reference Panel

In our derivation, $\mathbf{X} = [\mathbf{G} \quad \mathbf{G} \times \mathbf{T}]$

$$\mathbf{D} = \mathbf{X}'\mathbf{X}/n = \begin{bmatrix} \text{cor}(\mathbf{G}, \mathbf{G}) & \text{cor}(\mathbf{G}, \mathbf{G} \times \mathbf{T}) \\ \text{cor}(\mathbf{G} \times \mathbf{T}, \mathbf{G}) & \text{cor}(\mathbf{G} \times \mathbf{T}, \mathbf{G} \times \mathbf{T}) \end{bmatrix}.$$

With the information of (1) reference panel $\mathbf{G}$; (2) mean and variance of treatment factor $\mu_{\mathbf{T}}$ and $\sigma^2_{\mathbf{T}}$,

$$\begin{aligned} \text{cor}(G_i \times T, G_j) &= \frac{\mu_{\mathbf{T}} \sigma_{ij}}{\sqrt{(\mu^2_{\mathbf{T}} \sigma^2_i + 4 f^2_i \sigma^2_{\mathbf{T}} + \sigma^2_i \sigma^2_{\mathbf{T}}) \sigma^2_j}}, \\ \text{cor}(G_i \times T, G_j \times T) &= \frac{(\mu^2_{\mathbf{T}} + \sigma^2_{\mathbf{T}}) \sigma_{ij} + 4 \sigma^2_{\mathbf{T}} f_i f_j}{\sqrt{(\mu^2_{\mathbf{T}} \sigma^2_i + 4 f^2_i \sigma^2_{\mathbf{T}} + \sigma^2_i \sigma^2_{\mathbf{T}})(\mu^2_{\mathbf{T}} \sigma^2_j + 4 f^2_j \sigma^2_{\mathbf{T}} + \sigma^2_j \sigma^2_{\mathbf{T}})}}. \end{aligned} \tag{S.1}$$

where $\text{cov}(G_i, G_j) = \sigma_{ij}$, $\text{var}(G_i) = \sigma^2_i$, and f_i, f_j denote the minor allele frequency (MAF) of SNP i and j, respectively.

D.1. Difference between cor$(G_i \times T, G_j)$ and cor$(G_i, G_j \times T)$

In practice, we approximated $\text{cor}(\text{G} \times \text{T}, \text{G}) \approx \text{cor}(\text{G}, \text{G} \times \text{T})$ in the bottom-left block and top-right block of $\mathbf{D}$, which can reduce the computational cost of PRS-PGx-Bayes function. We can prove that such approximation held with the maximal difference smaller than 0.066.

Let f_i be the MAF of G_i. Under Hardy-Weinberg equilibrium, $\mu_i = \text{E}[G_i] = 2f_i$, $\sigma^2_i = \text{var}(G_i) = 2f_i(1 - f_i)$. We use subscript j and T for G_j and T, respectively. Further assume T and G_i (or G_j) are independent. First note that,

$$\text{cov}(G_i T, G_j) = \text{cov}(G_j T, G_i) = \text{E}[G_i G_j T] - \text{E}[G_i]\text{E}[G_j]\text{E}[T] = \sigma_{ij} \mu_T,$$

where σ_{ij} is the covariance between G_i and G_j.

To calculate the correlation, by definition,

$$\mathrm{cor}(G_iT, G_j) = \frac{\sigma_{ij}\mu_T}{\sqrt{\mathrm{var}(G_iT)\mathrm{var}(G_j)}},$$
$$\mathrm{cor}(G_jT, G_i) = \frac{\sigma_{ij}\mu_T}{\sqrt{\mathrm{var}(G_jT)\mathrm{var}(G_i)}},$$

where

$$\mathrm{var}(G_iT)\mathrm{var}(G_j) = \left(\sigma_i^2\sigma_T^2 + \sigma_i^2\mu_T^2 + \sigma_T^2\mu_i^2\right)\sigma_j^2,$$
$$\mathrm{var}(G_jT)\mathrm{var}(G_i) = \left(\sigma_j^2\sigma_T^2 + \sigma_j^2\mu_T^2 + \sigma_T^2\mu_j^2\right)\sigma_i^2.$$

This is due to the fact that $\mathrm{var}(XY) = \mathrm{var}(X)\mathrm{var}(Y) + \mathrm{var}(X)(\mathrm{E}[Y])^2 + \mathrm{var}(Y)(\mathrm{E}[X])^2$ if X and Y are independent.

Simplify to get

$$\begin{aligned}\mathrm{cor}(G_iT, G_j) - \mathrm{cor}(G_jT, G_i) &= \frac{\sigma_{ij}\mu_T}{\sqrt{\mathrm{var}(G_iT)\mathrm{var}(G_j)}} - \frac{\sigma_{ij}\mu_T}{\sqrt{\mathrm{var}(G_jT)\mathrm{var}(G_i)}} \\ (\text{let } \mu_T = 1/2) &= \frac{\sigma_{ij}}{\sigma_j\sqrt{\sigma_i^2 + \sigma_i^2 + \mu_i^2}} - \frac{\sigma_{ij}}{\sigma_i\sqrt{\sigma_j^2 + \sigma_j^2 + \mu_j^2}} \\ &= \mathrm{cor}(G_i, G_j)\left(\frac{1}{\sqrt{2 + \mu_i^2/\sigma_i^2}} - \frac{1}{\sqrt{2 + \mu_j^2/\sigma_j^2}}\right) \\ &= \mathrm{cor}(G_i, G_j)(\sqrt{1 - f_i} - \sqrt{1 - f_j})/\sqrt{2}.\end{aligned}$$

Without loss of generality, assume $0 < f_i \leq f_j \leq 0.5$. Follow the deduction in VanLiere and Rosenberg (2008)[3], it can be shown that

$$-\sqrt{r_j}\sqrt{r_i} \leq \mathrm{cor}(G_i, G_j) \leq \sqrt{r_i}/\sqrt{r_j},$$

where $r_i = f_i/(1 - f_i)$, $r_j = f_j/(1 - f_j)$. By Lagrangian multipliers, we can show that the absolute difference $|\mathrm{cor}(G_iT, G_j) - \mathrm{cor}(G_jT, G_i)| \leq 0.06626$ where the maximum is attained at the boundary $f_j = 0.5$, $f_i = 1 - \sqrt[3]{0.5} \approx 0.2063$ and $\mathrm{cor}(G_i, G_j) = \pm\sqrt{\sqrt[3]{2} - 1} \approx \pm 0.5098$.

To further evaluate the impact of such approximation on the final results, we compared predictive performances of PRS-PGx-Bayes with and without approximation in the simulation studies, where simulation settings remained the same as described in Fig. 2 in the main context (i.e., the heritability was fixed at 0.3, the traning sample size was 3000, and $\psi/\xi = 1$). Supplementary Figure 14 suggested that there was very little difference in terms of predictive performances when the approximation was used.

D.2. Several special cases of equation (S.1)

Now we consider several special cases of equation (S.1):

1. SNP i and SNP j ($i \neq j$) are independent (i.e., $\sigma_{ij} = 0$)

$$\mathrm{cor}(G_i \times T, G_j) = 0,\ i \neq j,$$

$$\mathrm{cor}(G_i \times T, G_j \times T) = \frac{4\sigma_{\mathbf{T}}^2 f_i f_j}{\sqrt{(\mu_{\mathbf{T}}^2\sigma_i^2 + 4f_i^2\sigma_{\mathbf{T}}^2 + \sigma_i^2\sigma_{\mathbf{T}}^2)(\mu_{\mathbf{T}}^2\sigma_j^2 + 4f_j^2\sigma_{\mathbf{T}}^2 + \sigma_j^2\sigma_{\mathbf{T}}^2)}},\ i \neq j.$$

2. SNP i and SNP j ($i \neq j$) are independent (i.e., $\sigma_{ij} = 0$); assume $T \sim \mathrm{Binomial}(1, p)$ (i.e., $\mu_T = p,\ \sigma_T^2 = p(1-p)$), since $G_i \sim \mathrm{Binomial}(2, f_i)$ and $G_j \sim \mathrm{Binomial}(2, f_j)$ (i.e., $\sigma_i^2 = 2f_i(1-f_i),\ \sigma_j^2 = 2f_j(1-f_j)$), we have

$$\mathrm{cor}(G_i \times T, G_j) = 0,\ i \neq j,$$

$$\mathrm{cor}(G_i \times T, G_j \times T) = \frac{2(1-p)f_i f_j}{\sqrt{f_i f_j (f_i - 2pf_i + 1)(f_j - 2pf_j + 1)}},\ i \neq j.$$

Further assume $p = 0.5$ (most typical randomized clinical trial):

$$\mathrm{cor}(G_i \times T, G_j) = 0,\ i \neq j,$$

$$\mathrm{cor}(G_i \times T, G_j \times T) = \sqrt{f_i f_j},\ i \neq j.$$

3. SNP i and SNP j ($i \neq j$) are independent (i.e., $\sigma_{ij} = 0$) and $T \equiv 1$.

$$\mathrm{cor}(G_i \times T, G_j) = 0,\ i \neq j,$$

$$\mathrm{cor}(G_i \times T, G_j \times T) = 0,\ i \neq j.$$

E. Posterior Distributions

Consider the following Bayesian regression model of n samples and m SNPs:

$$
\begin{aligned}
&\mathbf{Y} = \mathbf{G}\beta + (\mathbf{G} \times \mathbf{T})\alpha + \epsilon,\ \epsilon \sim \mathrm{N}(0, \sigma^2),\ p(\sigma^2) \propto (\sigma^2)^{-1} \\
&(\beta_j,\ \alpha_j) \sim \mathrm{N}(0, \frac{\sigma^2}{n}\mathbf{M}_j),\ \mathbf{M}_j = \begin{bmatrix} \psi_j & \rho_j\sqrt{\psi_j\xi_j} \\ \rho_j\sqrt{\psi_j\xi_j} & \xi_j \end{bmatrix},\ j = 1, \cdots, m \\
&\mathbf{M}_j \sim \mathrm{W}^{-1}(B_j,\ 2v+1),\ B_j = 4v \begin{bmatrix} \delta_j & 0 \\ 0 & \lambda_j \end{bmatrix},\ \delta_j \sim \mathrm{G}(b_1,\ \phi),\ \lambda_j \sim \mathrm{G}(b_2,\ \phi)
\end{aligned}
$$

E.1. The posterior distributions of b and σ^2

$$
\begin{aligned}
&\mathbf{b}|\mathbf{Y} \sim \mathrm{N}(\mu, \Sigma),\ \text{where } \mu = (\mathbf{D} + \Omega^{-1})^{-1}\widehat{\mathbf{b}}, \Sigma = \frac{\sigma^2}{n}(\mathbf{D} + \Omega^{-1})^{-1} \\
&\sigma^2|\mathbf{Y} \sim \mathrm{iG}\left(m + \frac{n}{2},\ \frac{n}{2}[\mathbf{b}'(\mathbf{D} + \Omega^{-1})\mathbf{b} + 1 - 2\widehat{\mathbf{b}}'\mathbf{b}]\right),\ \text{assume } \frac{\mathbf{Y}'\mathbf{Y}}{n} = 1 \text{ and } \widehat{\mathbf{b}} = \frac{\mathbf{X}'\mathbf{Y}}{n}
\end{aligned}
$$

where

$$
\begin{aligned}
&\mathbf{D} = \mathbf{X}'\mathbf{X}/n,\ \text{ and } \Omega = \begin{bmatrix} \Psi & P \\ P & \Xi \end{bmatrix}, \\
&\Psi = \mathrm{diag}(\phi\psi_j),\ \ \Xi = \mathrm{diag}(\phi\xi_j),\ \ P = \mathrm{diag}(\phi\rho_j\sqrt{\psi_j\xi_j}).
\end{aligned}
$$

To better understand how effect sizes are shrunk, we now provide special cases of $\mathrm{E}[\beta_j|\widehat{\beta}_j]$ and $\mathrm{E}[\alpha_j|\widehat{\alpha}_j]$ when $\mu_T = 0.5$. Specifically, under the assumptions that $\sigma_{ij} \equiv 0$, $i \neq j$, MAF $0 < f_j \equiv f \leq 0.5$, $\rho_j \equiv 0$ and $\mu_T = 0.5$, we can write

$$
D = \begin{bmatrix} \mathbf{I} & \sqrt{(1-f)/2}\mathbf{I} \\ \sqrt{(1-f)/2}\mathbf{I} & (1-f)\mathbf{I} \end{bmatrix} + \mathbf{g}\mathbf{g}',
$$

where $\mathbf{g} = [\mathbf{0}' \quad \sqrt{f}\mathbf{e}']'$, $\mathbf{e}$ is an $m \times 1$ vector of 1, $\mathbf{0}$ is an $m \times 1$ vector of 0.

$$\Omega = \begin{bmatrix} diag\{\phi\psi_j\} & \mathbf{0} \\ \mathbf{0} & diag\{\phi\xi_j\} \end{bmatrix} \Longrightarrow \Omega^{-1} = \begin{bmatrix} diag\{\phi^{-1}\psi_j^{-1}\} & \mathbf{0} \\ \mathbf{0} & diag\{\phi^{-1}\xi_j^{-1}\} \end{bmatrix}.$$

Thus we can write

$$D + \Omega^{-1} = A + \mathbf{g}\mathbf{g}',$$

where

$$A = \begin{bmatrix} diag\{1 + \phi^{-1}\psi_j^{-1}\} & diag\{\sqrt{(1-f)/2}\} \\ diag\{\sqrt{(1-f)/2}\} & diag\{1 - f + \phi^{-1}\xi_j^{-1}\} \end{bmatrix}.$$

To simplify notations, we write $t_j = 1+\phi^{-1}\psi_j^{-1}$, $s_j = 1-f+\phi^{-1}\xi_j^{-1}$ and $c_j = c = \sqrt{(1-f)/2}$, $j = 1, ..., m$. Then

$$A = \begin{bmatrix} diag\{t_j\} & diag\{c\} \\ diag\{c\} & diag\{s_j\} \end{bmatrix}.$$

By Sherman–Morrison formula, we have

$$(D + \Omega^{-1})^{-1} = A^{-1} - \frac{A^{-1}\mathbf{g}\mathbf{g}'A^{-1}}{1 + \mathbf{g}'A^{-1}\mathbf{g}}.$$

Next, we will compute each component

$$A^{-1} = \begin{bmatrix} diag\{\frac{s_j}{s_j t_j - c^2}\} & diag\{\frac{-c}{s_j t_j - c^2}\} \\ diag\{\frac{-c}{s_j t_j - c^2}\} & diag\{\frac{t_j}{s_j t_j - c^2}\} \end{bmatrix},$$

$$A^{-1}\mathbf{g} = \begin{bmatrix} diag\{\frac{s_i}{s_j t_j - c^2}\} & diag\{\frac{-c}{s_j t_j - c^2}\} \\ diag\{\frac{-c}{s_j t_j - c^2}\} & diag\{\frac{t_j}{s_j t_j - c^2}\} \end{bmatrix} \begin{bmatrix} \mathbf{0} \\ \sqrt{f}\mathbf{e} \end{bmatrix} = \begin{bmatrix} \begin{bmatrix} -\sqrt{f}\frac{c}{s_1 t_1 - c^2} \\ \cdots \\ -\sqrt{f}\frac{c}{s_m t_m - c^2} \end{bmatrix} \\ \begin{bmatrix} \sqrt{f}\frac{t_1}{s_1 t_1 - c^2} \\ \cdots \\ \sqrt{f}\frac{t_m}{s_m t_m - c^2} \end{bmatrix} \end{bmatrix},$$

$$A^{-1}\mathbf{g}\mathbf{g}'A^{-1} = \begin{bmatrix} \begin{bmatrix} -\sqrt{f}\frac{c}{s_1t_1-c^2} \\ ... \\ -\sqrt{f}\frac{c}{s_mt_m-c^2} \end{bmatrix} \\ \begin{bmatrix} \sqrt{f}\frac{t_1}{s_1t_1-c^2} \\ ... \\ \sqrt{f}\frac{t_m}{s_mt_m-c^2} \end{bmatrix} \end{bmatrix} \begin{bmatrix} \begin{bmatrix} -\sqrt{f}\frac{c}{s_1t_1-c^2} \\ ... \\ -\sqrt{f}\frac{c}{s_mt_m-c^2} \end{bmatrix} \\ \begin{bmatrix} \sqrt{f}\frac{t_1}{s_1t_1-c^2} \\ ... \\ \sqrt{f}\frac{t_m}{s_mt_m-c^2} \end{bmatrix} \end{bmatrix}' = \begin{bmatrix} f(\frac{c^2}{(s_it_i-c^2)(s_jt_j-c^2)})_{ij} & -f(\frac{ct_j}{(s_it_i-c^2)(s_jt_j-c^2)})_{ij} \\ -f(\frac{ct_i}{(s_it_i-c^2)(s_jt_j-c^2)})_{ij} & f(\frac{t_it_j}{(s_it_i-c^2)(s_jt_j-c^2)})_{ij} \end{bmatrix},$$

$$\mathbf{g}'A^{-1}\mathbf{g} = f\sum_{j=1}^{m} \frac{t_j}{s_jt_j - c^2}.$$

Finally,

$$A^{-1}\hat{b} = \begin{bmatrix} diag\{\frac{s_j}{s_jt_j-c^2}\} & diag\{\frac{-c}{s_jt_j-c^2}\} \\ diag\{\frac{-c}{s_jt_j-c^2}\} & diag\{\frac{t_j}{s_jt_j-c^2}\} \end{bmatrix} \begin{bmatrix} \hat{\beta} \\ \hat{\alpha} \end{bmatrix} = \begin{bmatrix} \frac{s_j\hat{\beta}_j - c\hat{\alpha}_j}{s_jt_j-c^2} \\ \frac{t_j\hat{\alpha}_j - c\hat{\beta}_j}{s_jt_j-c^2} \end{bmatrix},$$

$$\begin{aligned} A^{-1}\mathbf{g}\mathbf{g}'A^{-1}\hat{b} &= \begin{bmatrix} f(\frac{c^2}{(s_it_i-c^2)(s_jt_j-c^2)})_{ij} & -f(\frac{ct_j}{(s_it_i-c^2)(s_jt_j-c^2)})_{ij} \\ -f(\frac{ct_i}{(s_it_i-c^2)(s_jt_j-c^2)})_{ij} & f(\frac{t_it_j}{(s_it_i-c^2)(s_jt_j-c^2)})_{ij} \end{bmatrix} \begin{bmatrix} \hat{\beta} \\ \hat{\alpha} \end{bmatrix} \\ &= \begin{bmatrix} f\frac{c}{s_jt_j-c^2}\sum_{i=1}^m \frac{c}{s_it_i-c^2}\hat{\beta}_i - f\frac{c}{s_jt_j-c^2}\sum_{i=1}^m \frac{t_i}{s_it_i-c^2}\hat{\alpha}_i \\ f\frac{t_j}{s_jt_j-c^2}\sum_{i=1}^m \frac{t_i}{s_it_i-c^2}\hat{\alpha}_i - f\frac{t_j}{s_jt_j-c^2}\sum_{i=1}^m \frac{c}{s_it_i-c^2}\hat{\beta}_i \end{bmatrix}, \end{aligned}$$

$$\begin{aligned} \mathrm{E}[\beta_j|\hat{\beta}_j] &= \frac{s_j\hat{\beta}_j - c\hat{\alpha}_j}{s_jt_j - c^2} + \frac{1}{1+\mathbf{g}'A^{-1}\mathbf{g}}\left(f\frac{c}{s_jt_j-c^2}\sum_{i=1}^m \frac{c}{s_it_i-c^2}\hat{\beta}_i - f\frac{c}{s_jt_j-c^2}\sum_{i=1}^m \frac{t_i}{s_it_i-c^2}\hat{\alpha}_i\right) \\ &= \frac{s_j\hat{\beta}_j - c\hat{\alpha}_j}{s_jt_j - c^2} + \frac{f\frac{c}{s_jt_j-c^2}}{1+\mathbf{g}'A^{-1}\mathbf{g}}\left(\sum_{i=1}^m \frac{c}{s_it_i-c^2}\hat{\beta}_i - \sum_{i=1}^m \frac{t_i}{s_it_i-c^2}\hat{\alpha}_i\right) \\ &= \frac{s_j\hat{\beta}_j - c\hat{\alpha}_j}{s_jt_j - c^2} + \frac{f\frac{c}{s_jt_j-c^2}}{1+f\sum_{i=1}^m \frac{t_i}{s_it_i-c^2}}\left(\sum_{i=1}^m \frac{c\hat{\beta}_i - t_i\hat{\alpha}_i}{s_it_i-c^2}\right) \\ &= \frac{\hat{\beta}_j - \frac{c}{s_j}\hat{\alpha}_j}{t_j - c^2/s_j} + \frac{f\frac{c}{s_jt_j-c^2}}{1+f\sum_{i=1}^m \frac{t_i}{s_it_i-c^2}}\left(\sum_{i=1}^m \frac{c\hat{\beta}_i - t_i\hat{\alpha}_i}{s_it_i-c^2}\right), \end{aligned}$$

$$
\begin{aligned}
\mathrm{E}[\alpha_j|\hat{\alpha}_j] &= \frac{t_j\hat{\alpha}_j - c\hat{\beta}_j}{s_j t_j - c^2} + \frac{1}{1+\mathbf{g}'A^{-1}\mathbf{g}}\left(f\frac{t_j}{s_j t_j - c^2}\sum_{i=1}^{m}\frac{t_i}{s_i t_i - c^2}\hat{\alpha}_i - f\frac{t_j}{s_j t_j - c^2}\sum_{i=1}^{m}\frac{c}{s_i t_i - c^2}\hat{\beta}_i\right) \\
&= \frac{t_j\hat{\alpha}_j - c\hat{\beta}_j}{s_j t_j - c^2} + \frac{f\frac{t_j}{s_j t_j - c^2}}{1+\mathbf{g}'A^{-1}\mathbf{g}}\left(\sum_{i=1}^{m}\frac{t_i}{s_i t_i - c^2}\hat{\alpha}_i - \sum_{i=1}^{m}\frac{c}{s_i t_i - c^2}\hat{\beta}_i\right) \\
&= \frac{t_j\hat{\alpha}_j - c\hat{\beta}_j}{s_j t_j - c^2} + \frac{f\frac{t_j}{s_j t_j - c^2}}{1+f\sum_{i=1}^{m}\frac{t_i}{s_i t_i - c^2}}\left(\sum_{i=1}^{m}\frac{t_i\hat{\alpha}_i - c\hat{\beta}_i}{s_i t_i - c^2}\right) \\
&= \frac{\hat{\alpha}_j - \frac{c}{t_j}\hat{\beta}_j}{s_j - c^2/t_j} + \frac{f\frac{t_j}{s_j t_j - c^2}}{1+f\sum_{i=1}^{m}\frac{t_i}{s_i t_i - c^2}}\left(\sum_{i=1}^{m}\frac{t_i\hat{\alpha}_i - c\hat{\beta}_i}{s_i t_i - c^2}\right).
\end{aligned}
$$

Further consider a small MAF for all markers (i.e., $f \to 0$), we have:

$$
\begin{aligned}
\mathrm{E}[\beta_j|\hat{\beta}_j] &\approx \frac{\hat{\beta}_j - \frac{c}{s_j}\hat{\alpha}_j}{t_j - c^2/s_j}, \\
\mathrm{E}[\alpha_j|\hat{\alpha}_j] &\approx \frac{\hat{\alpha}_j - \frac{c}{t_j}\hat{\beta}_j}{s_j - c^2/t_j}.
\end{aligned}
$$

E.2. The posterior distribution of $\mathbf{M}_j$

$$
\mathbf{M}_j|\beta_j, \alpha_j, \delta_j, \lambda_j, \sigma^2 \sim \mathrm{W}^{-1}(B_j + A_j, 2v+2), \;\; A_j = \frac{n}{\sigma^2}\begin{bmatrix} \beta_j^2 & \beta_j\alpha_j \\ \beta_j\alpha_j & \alpha_j^2 \end{bmatrix}
$$

which is to say marginally $\psi_j \sim \mathrm{iG}\left(v + 1/2, \delta_j + \frac{n}{2\sigma^2}\beta_j^2\right)$, $\xi_j \sim \mathrm{iG}\left(v + 1/2, \lambda_j + \frac{n}{2\sigma^2}\alpha_j^2\right)$.

E.3. The posterior distributions of δ_j and λ_j

$$
\begin{aligned}
p(\delta_j, \lambda_j|\mathbf{M}_j) &\propto p(\mathbf{M}_j|\delta_j, \lambda_j)p(\delta_j, \lambda_j) \\
&\qquad \propto |B_j|^{(2v+1)/2} e^{-\frac{1}{2}\mathrm{tr}(B_j\mathbf{M}_j^{-1})}\delta^{b_1-1}e^{-\phi\delta_j}\lambda^{b_2-1}e^{-\phi\lambda_j} \\
&\Longrightarrow \delta_j|\mathbf{M}_j \sim \mathrm{G}(v + b_1 + 1/2, \phi + \frac{2v}{\psi_j(1-\rho_j^2)}) \\
&\qquad \lambda_j|\mathbf{M}_j \sim \mathrm{G}(v + b_2 + 1/2, \phi + \frac{2v}{\xi_j(1-\rho_j^2)}).
\end{aligned}
$$

F. PGx GWAS Summary Statistics

In PRS-PGx-Bayes, we assume that $X = [G \quad G \times T]$ and Y have been standardized. However, when it comes to the construction of PRS, we should use unstandardized coefficients as weights. To convert standardized coefficients to unstandardized ones, we can use the following formula:

$$b^{\text{unstandardized}} = b^{\text{standardized}} \times \frac{\text{sd}(Y)}{\text{sd}(X)}.$$

When $X = G$, we only need $\text{var}(G) = \sigma_G^2$. When $X = TG$, assuming the treatment assignment is independent of the genotype (which is true in RCT), we have

$$\begin{aligned}\text{var}(TG) &= (\text{E}[T])^2\text{var}(G) + (\text{E}[G])^2\text{var}(T) + \text{var}(T)\text{var}(G) \\ &= \mu_T^2\sigma_G^2 + \mu_G^2\sigma_T^2 + \sigma_T^2\sigma_G^2.\end{aligned}$$

Further, assuming $T \sim \text{Bernoulli}(\mu_T)$, we have

$$\sigma_T^2 = \mu_T(1 - \mu_T).$$

Assuming $G \sim \text{Binomial}(2,\ \text{MAF})$, where MAF denotes the minor allele frequency, we have

$$\mu_G = 2 \times \text{MAF}, \quad \sigma_G^2 = 2 \times \text{MAF} \times (1 - \text{MAF}).$$

Therefore, we only need μ_T and MAF to calculate the value of $\text{sd}(X)$.

According to the above discussion, the PGx GWAS summary statistics required by our proposed PRS-PGx-Bayes method as inputs should include

1. the SNP ID, MAF, and position;
2. the prognostic and predictive effect sizes ($\widehat{\beta}$, $\widehat{\alpha}$);
3. the 2df (joint G + G $\times$ T) two-sided test p-value;
4. the standard deviation of the drug response (sd(Y));

5. the mean of the treatment assignment (μ_T).

For the other two PRS-PGx methods (PRS-PGx-Unadj and PRS-PGx-CT), only the summary statistics in the first three items are needed as inputs.

G. Random Mating

Random mating is commonly used in pharmarcogenomics to increase sample size. By re-sampling at the haplotype level instead of the SNP level, the LD structure is essentially preserved[4]. Random mating is done in two steps:

1. Within each chromosome, for each individual, split his/her two DNA strands into two haplotypes (i.e., two vectors of nucleotides, one from each parent), and generate a reference pool of $2n$ haplotypes. Suppose there are total n patients in the trial.

2. To form the genotype of a new individual, two haplotypes are randomly sampled from the reference pool and are combined. Repeat the above procedure and check the relatedness between previous generated individuals and the new individual, until the desired number of patients, say $n^* > n$, is achieved.

Supplementary Figures

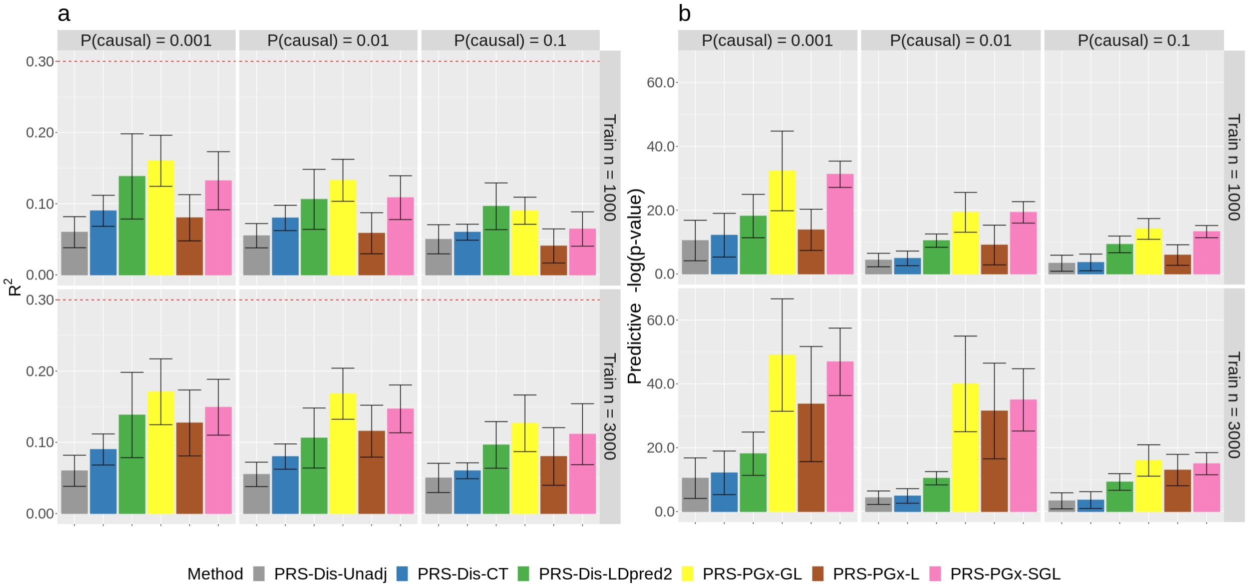

Supplementary Figure 1: The drug response prediction performance of the three disease PRS methods (PRS-Dis-Unadj, -CT, -LDpred2) and the three penalized regression PGx PRS methods (PRS-PGx-L, -GL, -SGL). The performance was assessed based on the simulated data, where heritability was fixed at 0.3, $\beta_T = 0$, and the SNP prognostic and predictive effect sizes jointly followed a bivariate normal distribution, with $\psi/\xi = 1$. The numbers of the causal variants for P(causal)=0.001, 0.01 and 0.1 were 5, 50 and 500, respectively. Data are presented as mean values +/- standard deviations (error bars) with 10,000 replications, where results were calculated from the testing sets. **a** Barplots showing the prediction accuracy, R^2, of S_{PGx}. **b** Barplots showing the predictive p-value for the two-sided $S_{pred} \times T$ interaction test.

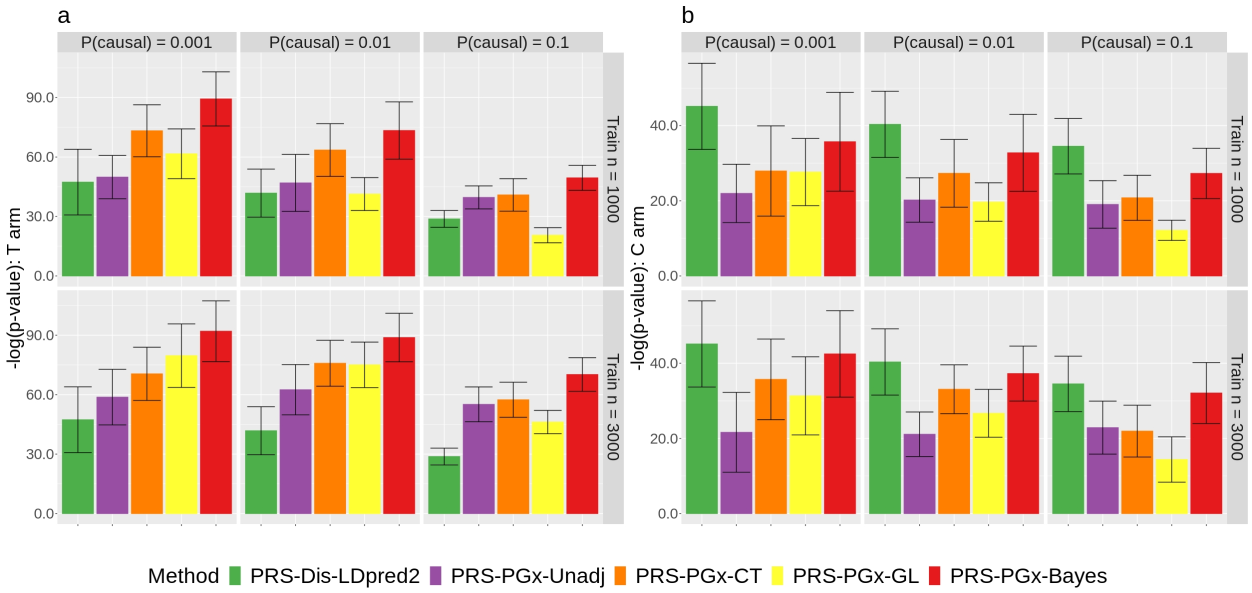

Supplementary Figure 2: Predictive performance of five polygenic prediction methods in the simulation studies, where heritability was fixed at 0.3, $\boldsymbol{\beta_T} = 0$, and $\boldsymbol{\psi/\xi} = 1$. The numbers of the causal variants for P(causal)=0.001, 0.01 and 0.1 were 5, 50 and 500, respectively. The tuning parameters were selected via cross-validation in the training data. Data are presented as mean values +/- standard deviations (error bars) with 10,000 replications, where results were calculated from the testing sets. P-values were assessed by the two-sided LRT of S_{PGx} from $Y \sim S_{PGx}$ under (**a**) treatment arm and (**b**) control arm, respectively.

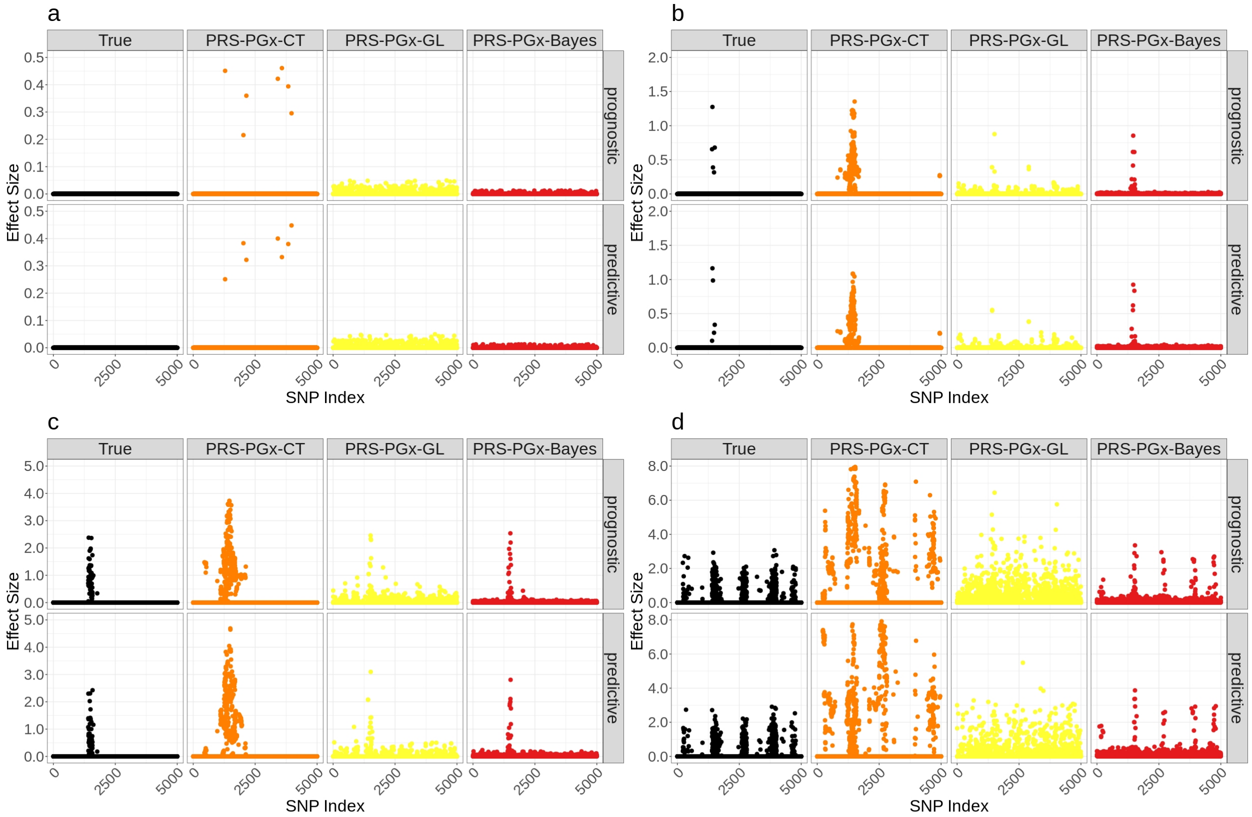

Supplementary Figure 3: Distributions of $(\widehat{\beta}, \widehat{\alpha})$ estimated by the PRS-PGx methods versus the true values of (β, α) under different genetic architectures. The response was simulated with $H^2 = 0.3$, $\beta_T = 0$, and $\psi/\xi = 1$. $(\widehat{\beta}, \widehat{\alpha})$ were estimated using all 5,661 patients in the IMPROVE-IT data. Hyper-parameters were determined by the 5-fold cross-validation. **a** No causal variant: P(causal) = 0. Hyper-parameters were chosen as p-value cutoff = 1e-03; penalty $\lambda = 0.5$; for PRS-PGx-Bayes, $\upsilon = 3$, $\phi^{1/2} = 0.0001$. **b** P(causal) = 0.001. Hyper-parameters were chosen as p-value cutoff = 1e-04; penalty $\lambda = 0.1$; for PRS-PGx-Bayes, $\upsilon = 2$, $\phi^{1/2} = 0.001$. **c** P(causal) = 0.01. Hyper-parameters were chosen as p-value cutoff = 1e-04; penalty $\lambda = 0.1$; for PRS-PGx-Bayes, $\upsilon = 4$, $\phi^{1/2} = 0.0005$. **d** P(causal) = 0.1. Hyper-parameters were chosen as p-value cutoff = 1e-03; penalty $\lambda = 0.07$; for PRS-PGx-Bayes, $\upsilon = 2$, $\phi^{1/2} = 0.001$.

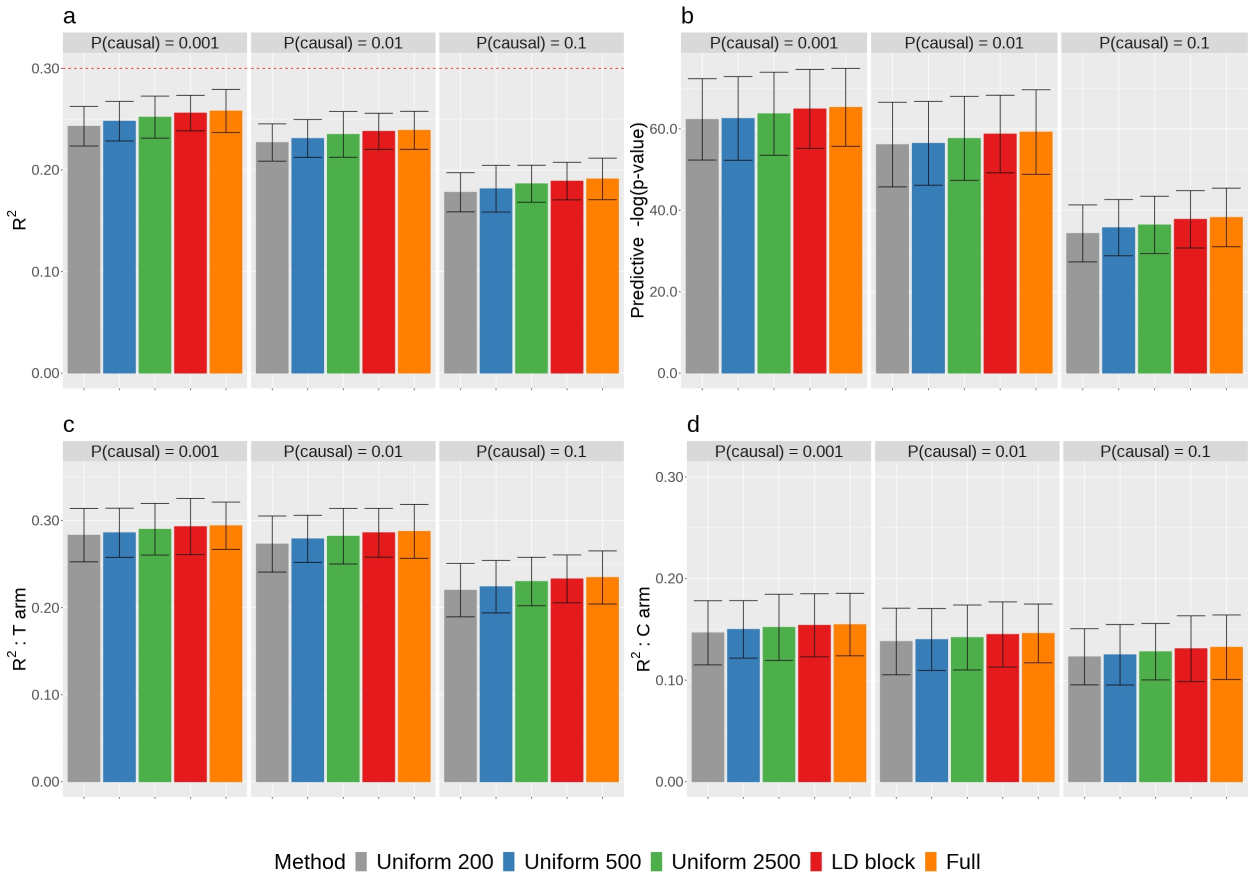

Supplementary Figure 4: Performance comparisons of PRS-PGx-Bayes method when carried out by different implementation strategies (based on LD blocks 31 and 32 on chromosome 19) in simulation studies, where heritability was fixed at 0.3, training sample size = 3,000, and $\psi/\xi = 1$. PRS-PGx-Bayes was carried out by the uniform block with size 200, 500, and 2,500, the LD block, and the full genotype data. The numbers of the causal variants for P(causal) = 0.001, 0.01 and 0.1 were 5, 50 and 500, respectively. The tuning parameters of PRS-PGx-Bayes were selected via cross-validation in the training data. Data are presented as mean values +/- standard deviations (error bars) with 10,000 replications, where results were calculated from the testing sets. The performance was assessed in terms of (**a**) prediction accuracy R^2 of S_{PGx} in two arms, (**b**) p-value for the two-sided $S_{pred} \times T$ interaction test, (**c**) R^2 of S_{PGx} under treatment arm, and (**d**) R^2 of S_{PGx} under control arm.

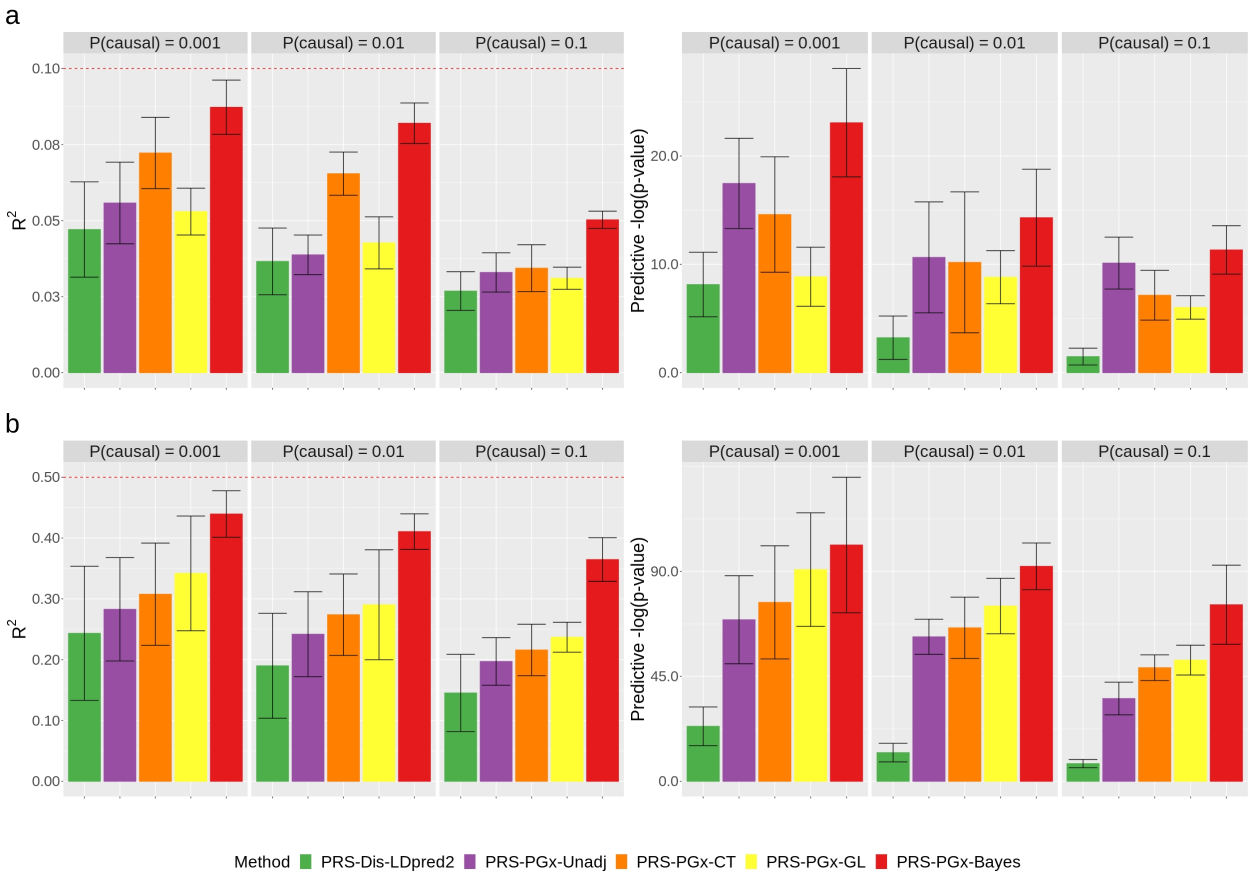

Supplementary Figure 5: The performance of the five methods based on the sensitivity analyses when the heritability was set a 0.1 or 0.5. The performance was assessed by the prediction accuracy R^2 and the predictive p-values from two-sided interaction tests in (**a**) $H^2 = 0.1$, and (**b**) $H^2 = 0.5$. The response was simulated with $\beta_T = 0$ and $\psi/\xi = 1$. The numbers of the causal variants for P(causal)=0.001, 0.01 and 0.1 were 5, 50 and 500, respectively. The training size was fixed as 3,000. Data are presented as mean values +/- standard deviations (error bars) with 10,000 replications, where results were calculated from the testing sets.

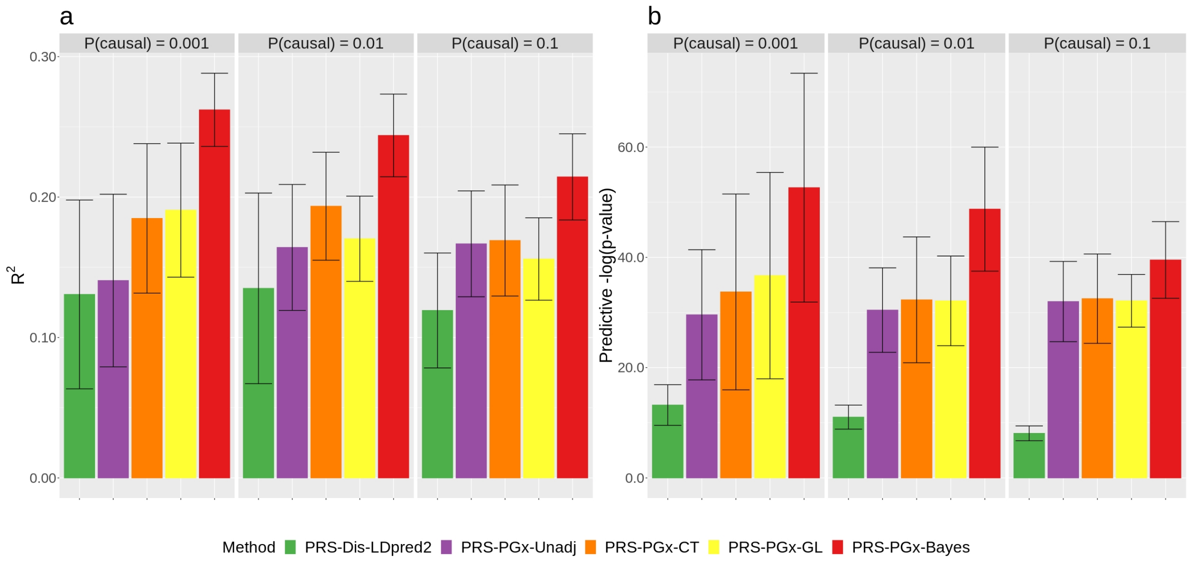

Supplementary Figure 6: The performance of the five methods based on the sensitivity analyses when the coefficient of treatment $\boldsymbol{\beta_T = 1}$. The heritability was fixed at 0.3, $\psi/\xi = 1$, and training size was equivalent to 3,000. The numbers of the causal variants for P(causal)=0.001, 0.01 and 0.1 were 5, 50 and 500, respectively. Data are presented as mean values +/- standard deviations (error bars) with 10,000 replications, where results were calculated from the testing sets. **a** The performance was assessed by the prediction accuracy R^2. **b** The performance was assessed by the predictive p-values from two-sided interaction tests, which measure methods' capabilities of capturing the predictive effect.

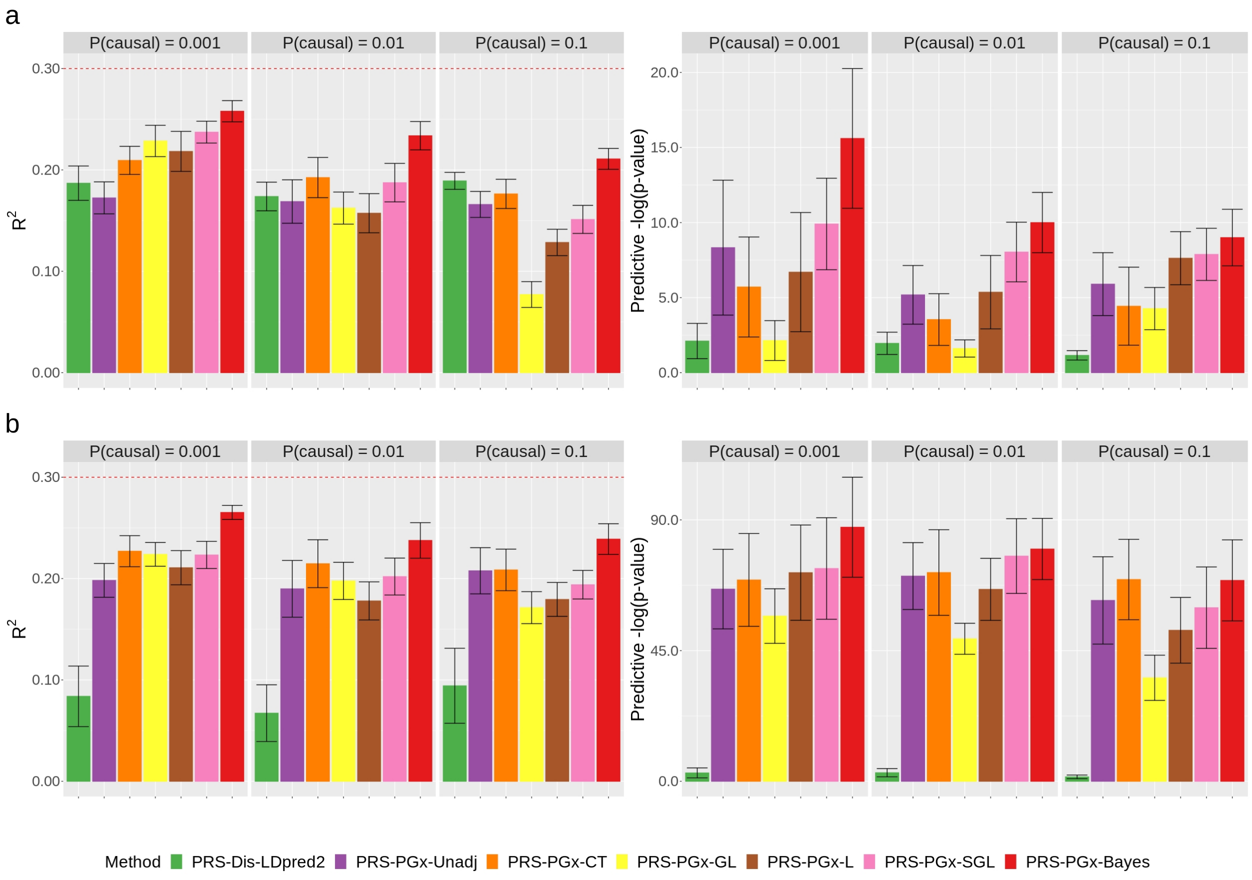

Supplementary Figure 7: The performance of the seven methods based on the sensitivity analyses when the prognostic and predictive effect sizes had different scales. The heritability was fixed at 0.3. $\beta_T = 0$. Training $n = 3{,}000$. The numbers of the causal variants for P(causal)=0.001, 0.01 and 0.1 were 5, 50 and 500, respectively. The performance was assessed by the prediction accuracy R^2 and the predictive p-value from two-sided interaction test. Data are presented as mean values +/- standard deviations (error bars) with 10,000 replications, where results were calculated from the testing sets. **a** $\psi/\xi = 16$, which means the prognostic effect was dominant to the predictive effect. **b** $\psi/\xi = 1/16$, which means the predictive effect was dominant to the prognostic effect.

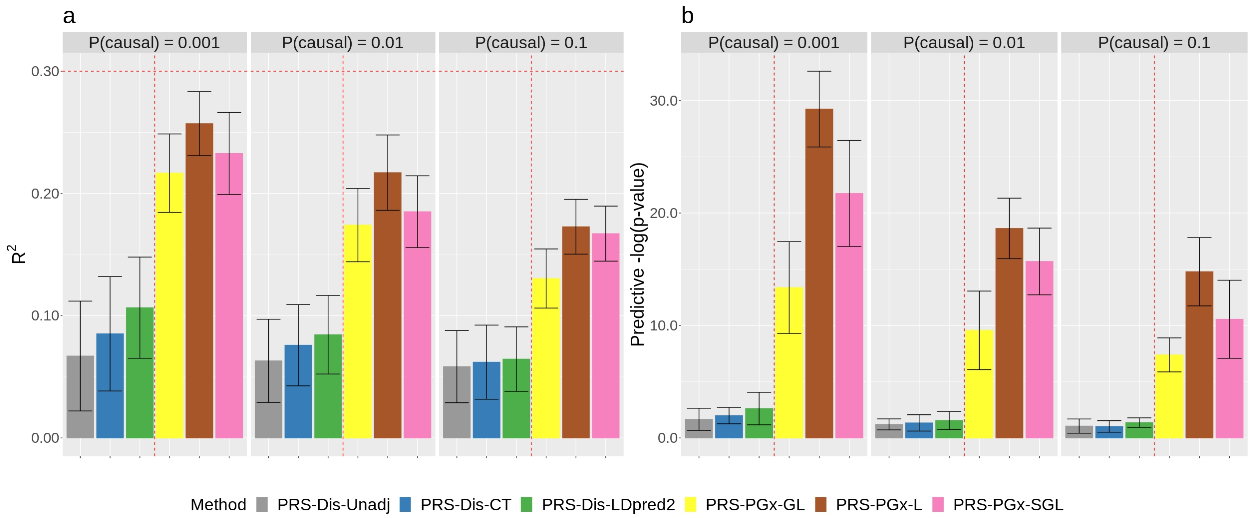

Supplementary Figure 8: The drug response prediction performance of the three disease PRS methods (PRS-Dis-Unadj, -CT, -LDpred2) and the three penalized regression PGx PRS methods (PRS-PGx-L, -GL, -SGL) when the SNP prognostic and predictive effect sizes were completely separated. The performance was assessed based on the simulated data and the heritability was fixed at 0.3. The numbers of the causal variants for P(causal)=0.001, 0.01 and 0.1 were 5, 50 and 500, respectively. The training sample size was set as 3,000. Data are presented as mean values +/- standard deviations (error bars) with 10,000 replications, where results were calculated from the testing sets. **a** Barplots showing the prediction accuracy, R^2. **b** Barplots showing the predictive p-value for the two-sided $S_{pred} \times T$ interaction test.

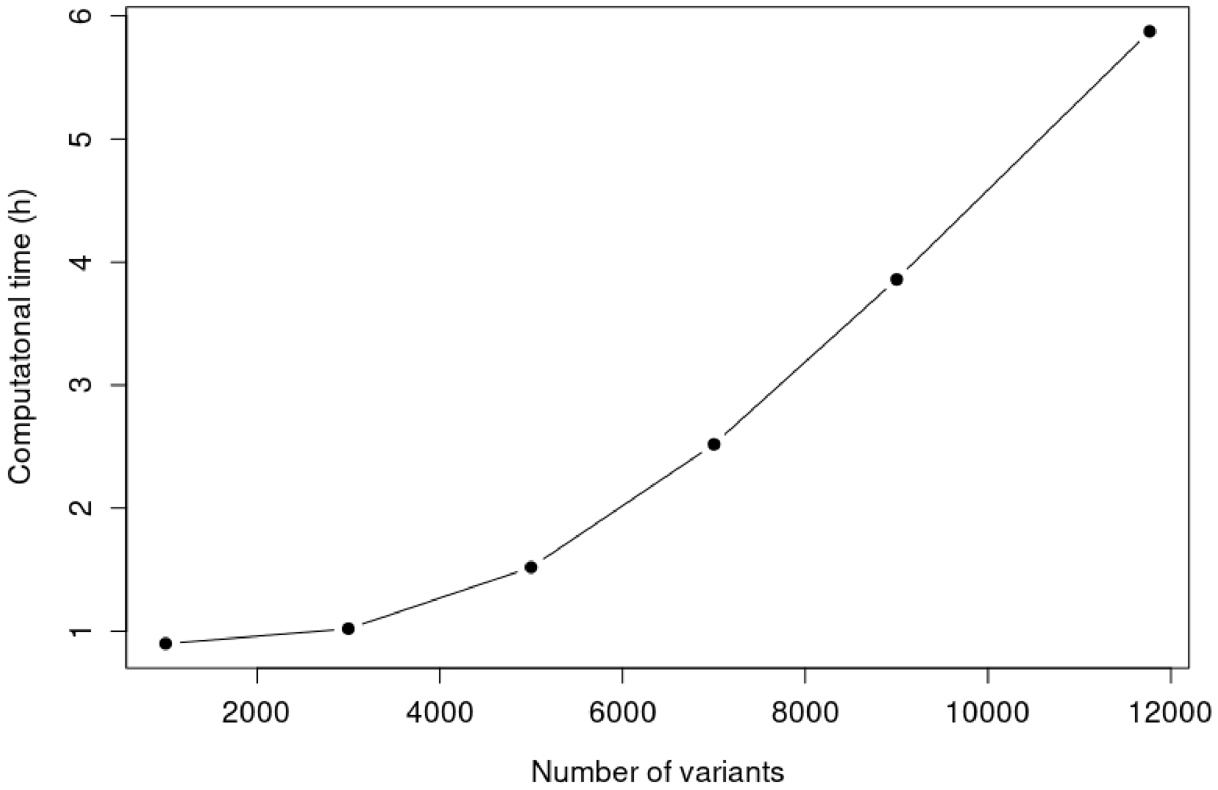

Supplementary Figure 9: Computational time on the largest LD block (chr 6, block 33) by running PRS-PGx-Bayes function with 1,000 MCMC iterations. Number of variants = 1,000, 3,000, 5,000, 7,000, 9,000, and 11,769 (whole LD block). The real genetic data was obtained from the IMPROVE-IT trial with the sample size of 5,661. The effect sizes and phenotype data were simulated with heritability fixed at 0.3, $\psi/\xi = 1$, and P(causal) = 0.01.

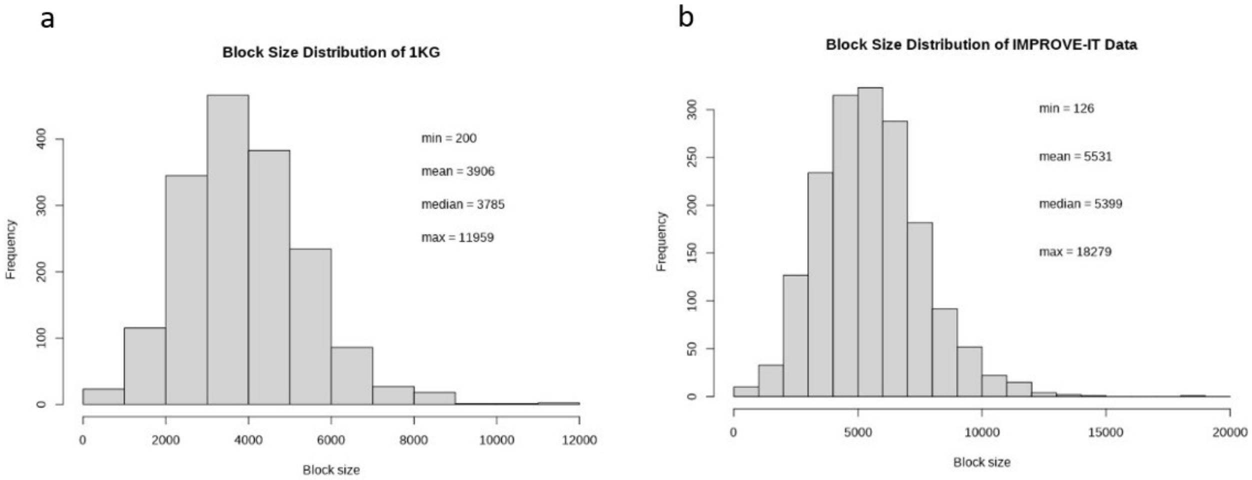

Supplementary Figure 10: Distribution of LD block sizes where SNPs are included in (a) 1KG, and (b) IMPROVE-IT. Summary statistics are also attached in the figure. The total number of independent LD blocks is 1,725 for European population.

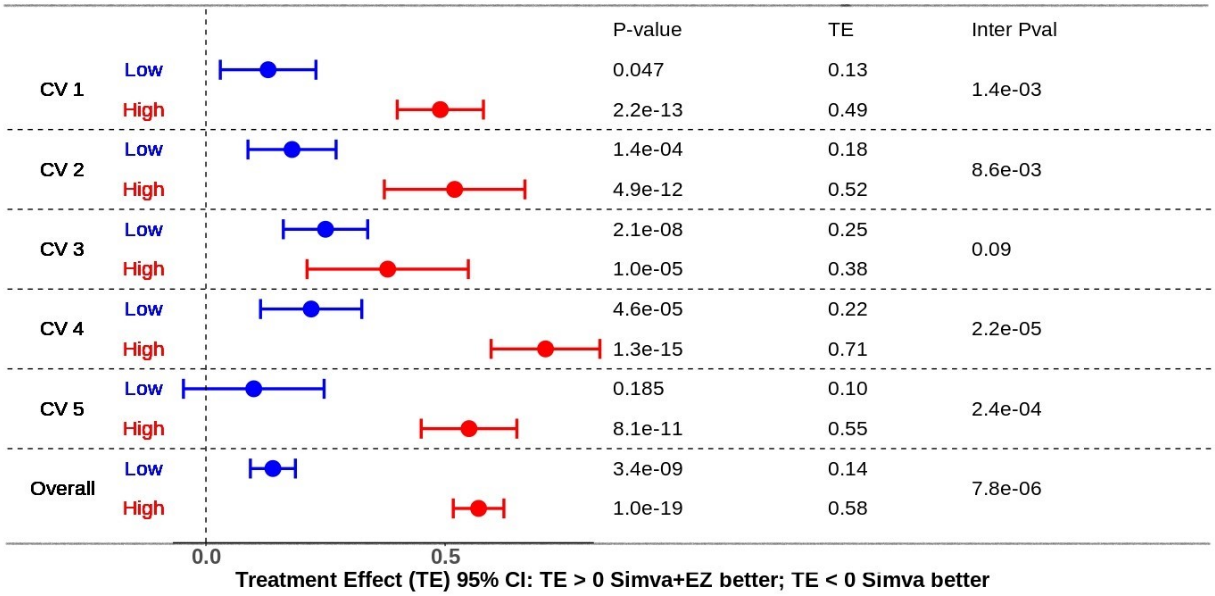

Supplementary Figure 11: Quantile plot of treatment effect in each of the five fold validation datasets and the full validation dataset from a nested cross-validation analysis of the IMPROVE-IT PGx GWAS data with total n = 5,661 unrelated European samples. Each dot stands for the observed Treatment Effect (TE), and each bar denotes the 95% Confidence Interval (CI). The low and high risk subgroups were determined by the predictive score of PRS-PGx-Bayes, and the optimal cutoff was decided via cross-validation: CV 1 (70%), CV 2 (40%), CV 3 (50%), CV 4 (30%), CV 5 (70%).

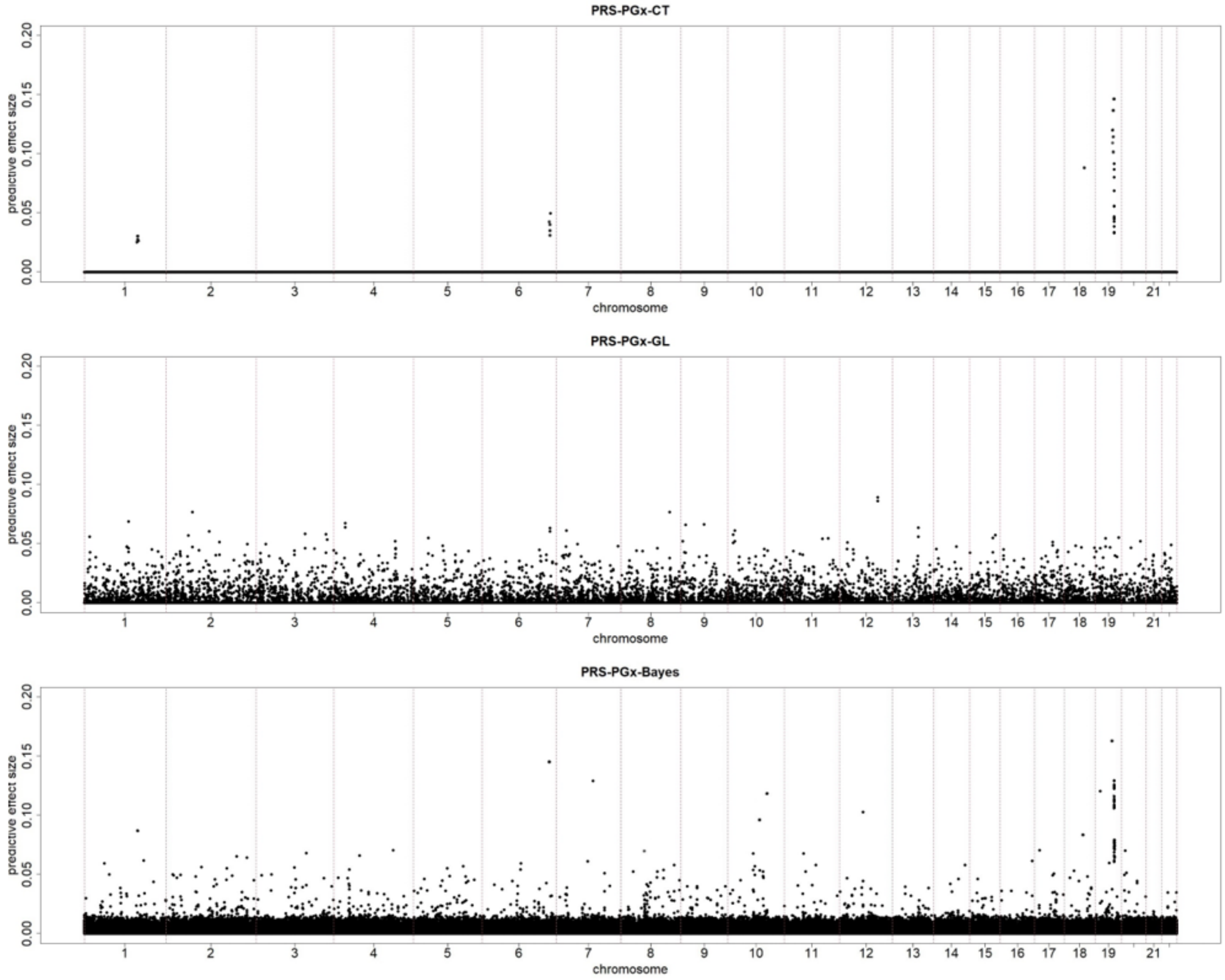

Supplementary Figure 12: Distributions of the predictive effect sizes across the whole genome. The predictive effect sizes were estimated by three PRS-PGx methods (PRS-PGx-CT, PRS-PGx-GL and PRS-PGx-Bayes) from the IMPROVE-IT PGx GWAS (summary statistics) data.

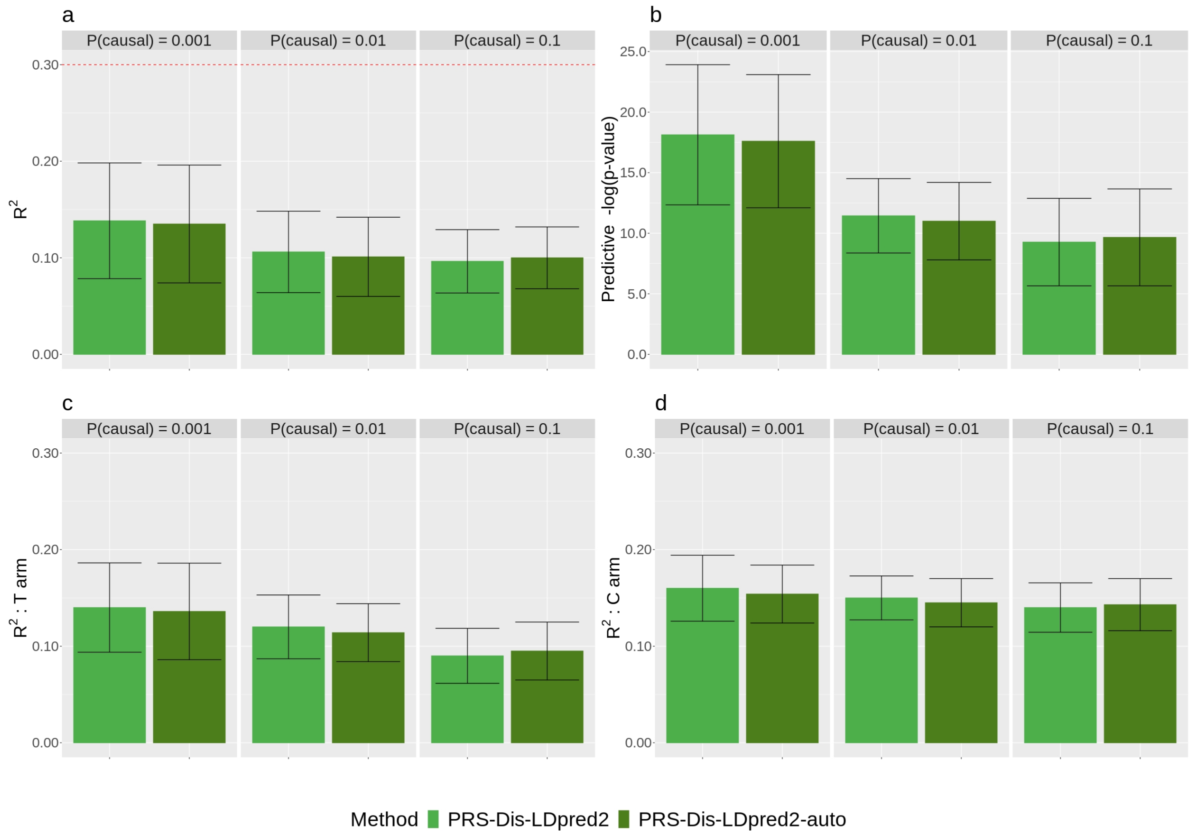

Supplementary Figure 13: Predictive performance of LDpred2-grid and LDpred2-auto methods in the simulation studies, where heritability was fixed at 0.3 and $\psi/\xi = 1$. The numbers of the causal variants for P(causal) = 0.001, 0.01 and 0.1 were 5, 50 and 500, respectively. The training sample size was 20,000. The tuning parameters of LDpred2-grid were selected via cross-validation in the training data. Data are presented as mean values +/- standard deviations (error bars) with 10,000 replications, where results were calculated from the testing sets. The performance was assessed in the testing set in terms of (**a**) prediction accuracy R^2 of S_{PGx} in two arms, (**b**) predictive p-value of the two-sided $S_{pred} \times T$ interaction test, (**c**) R^2 of S_{PGx} under treatment arm, and (**d**) R^2 of S_{PGx} under control arm.

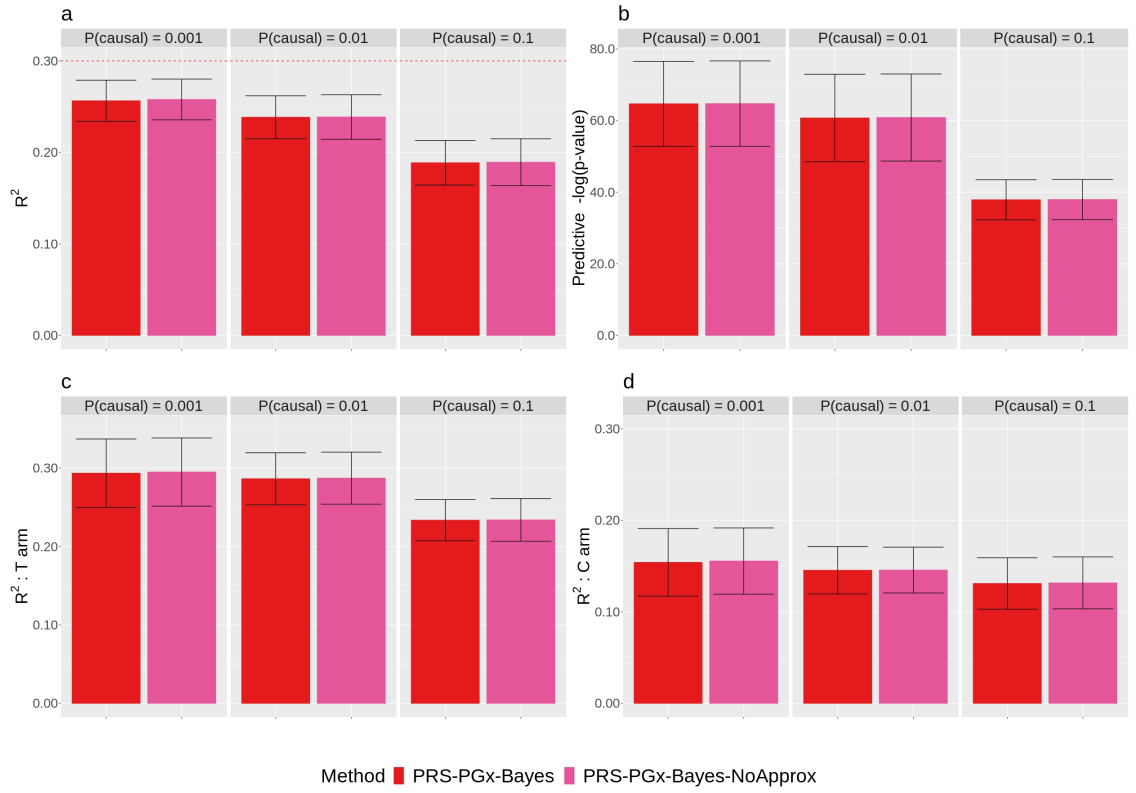

Supplementary Figure 14: Predictive performance of PRS-PGx-Bayes with or without approximation (i.e., $\mathrm{cor}(\mathbf{G} \times \mathbf{T}, \mathbf{G}) \approx \mathrm{cor}(\mathbf{G}, \mathbf{G} \times \mathbf{T})$) in the simulation studies, where heritability was fixed at 0.3 and $\psi/\xi = 1$. The numbers of the causal variants for P(causal) = 0.001, 0.01 and 0.1 were 5, 50 and 500, respectively. The training sample size was 3,000. Data are presented as mean values +/- standard deviations (error bars) with 10,000 replications, where results were calculated from the testing sets. The performance was assessed in terms of (**a**) prediction accuracy R^2 of S_{PGx} in two arms, (**b**) predictive p-value of the two-sided $S_{pred} \times T$ interaction test, (**c**) R^2 of S_{PGx} under treatment arm, and (**d**) R^2 of S_{PGx} under control arm.

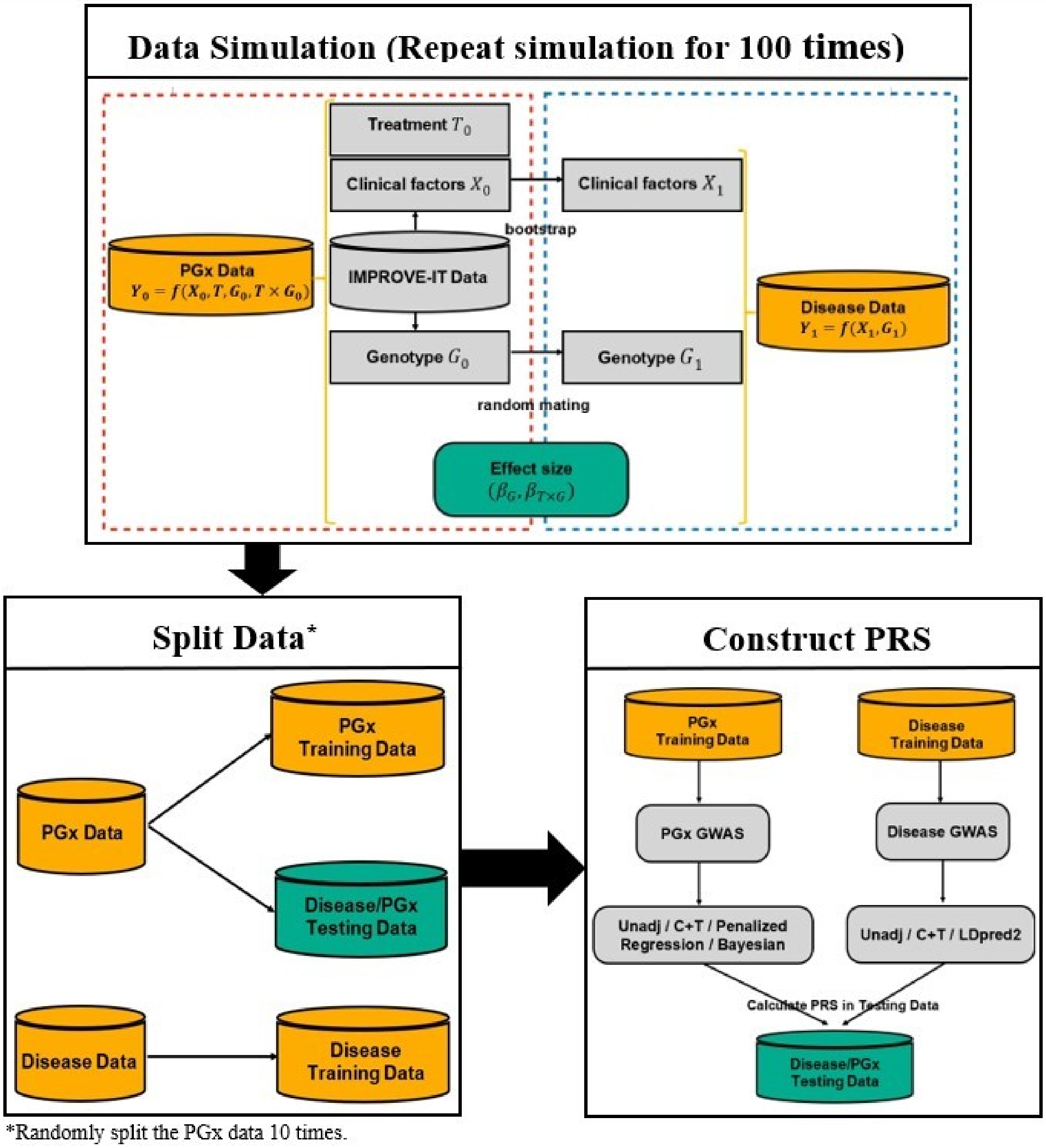

Supplementary Figure 15: Workflow of simulation studies. In Step 1, genotype, treatment and clinical factors were extracted from IMPROVE-IT PGx data. The prognostic and predictive effect sizes were simulated jointly as described in Section 4.5. The disease and PGx data were prepared for further analysis. In Step 2, PGx data were randomly split into the training and testing sets. In Step 3, the disease PRS and PGx PRS were constructed by using the PGx training data and disease summary statistics data, respectively. All the constructed PRSs were tested in the PGx testing data.

Supplementary Tables

Supplementary Table 1: Performance decreases of PRS-PGx-Bayes method carried out from using full genotype matrix (i.e., using LD blocks 31 and 32 jointly on chromosome 19) to using LD blocks (i.e., using LD blocks 31 and 32 separately on chromosome 19) in simulation studies. Heritability was fixed at 0.3, training sample size = 3000, and $\psi/\xi = 1$. The numbers of the causal variants for P(causal) = 0.001, 0.01 and 0.1 were 5, 50 and 500, respectively. The performance was assessed in terms of prediction accuracy R^2 of S_{PGx} in two arms, predictive p-value of two-sided $S_{pred} \times T$ interaction test, R^2 of S_{PGx} under treatment arm, and R^2 of S_{PGx} under control arm.

P(causal)	0.001	0.01	0.1
R^2	0.4%	0.7%	0.9%
Pred P-value	0.6%	0.6%	0.9%
R^2: T arm	0.3%	0.5%	0.8%
R^2: C arm	0.5%	0.7%	1.1%

Supplementary Table 2: Information of the top 20 SNPs ranked by their absolute predictive effect sizes ($\beta^{\text{Bayes}}_{\mathbf{G\times T}}$) estimated from PRS-PGx-Bayes. The last five columns (2-df (joint G + G $\times$ T) two-sided p-value, $\widehat{\beta}_{\mathbf{G}}$, main effect two-sided p-value, $\widehat{\beta}_{\mathbf{G\times T}}$, interaction effect two-sided p-value) were obtained from IMPROVE-IT PGx GWAS summary statistics. $\beta^{\text{Bayes}}_{\mathbf{G}}$ and $\beta^{\text{Bayes}}_{\mathbf{G\times T}}$ were estimated by PRS-PGx-Bayes. "– –" indicates that there is no reported trait of LDL Cholesterol associated with the corresponding SNP in Open Targets (https://genetics.opentargets.org).

SNP ID	Chr	Position	Gene	Reported Trait	$\beta^{\text{Bayes}}_{\mathbf{G}}$	$\beta^{\text{Bayes}}_{\mathbf{G\times T}}$	2-df P-value	$\widehat{\beta}_{\mathbf{G}}$	P-value (G)	$\widehat{\beta}_{\mathbf{G\times T}}$	P-value (G×T)
rs10455872	6	161010118	*LPA*	LDL Cholesterol	0.12	-0.14	8.5e-15	0.09	6.8e-15	-0.05	0.03
rs1761667	7	85555136	*CD36*	– –	0.004	0.13	0.33	0.002	0.694	0.02	0.149
rs61276248	10	120907615	*SFXN4*	– –	-0.05	0.11	2.3e-06	-0.03	1.5e-04	0.06	6.7e-04
rs76495189	12	78567518	*AC073571.1*	– –	-0.006	-0.10	0.28	-0.01	0.463	-0.05	0.161
rs78706454	19	8103088	*CCL25*	– –	0.002	0.12	1.5e-03	-0.01	0.33	0.05	5.2e-04
rs111371860	19	45345787	*NECTIN2*	LDL Cholesterol	-0.09	0.10	1.9e-10	-0.08	1.0e-10	0.04	0.083
rs283810	19	45388241	*AC011481.1*	LDL Cholesterol	-0.02	0.12	1.3e-11	-0.08	3.5e-10	0.08	0.001
rs61679753	19	45400747	*AC011481.1*	LDL Cholesterol	-0.24	0.15	3.5e-34	-0.19	1.5e-31	0.14	3.0e-05
rs769446	19	45408628	*APOE*	LDL Cholesterol	-0.003	0.10	8.7e-11	-0.07	1.3e-10	0.05	0.026
rs429358	19	45411941	*APOE*	LDL Cholesterol	0.10	0.12	1.6e-07	0.05	4.0e-08	-0.02	0.287
rs7412	19	45412079	*APOE*	LDL Cholesterol	-0.19	0.13	7.5e-52	-0.16	5.2e-50	0.08	1.5e-04
rs1065853	19	45413233	*AC011481.3*	LDL Cholesterol	-0.06	0.10	5.9e-52	-0.16	3.9e-50	0.08	1.6e-04
rs7256200	19	45415935	*APOC1*	– –	0.01	0.10	3.7e-06	0.05	1.3e-06	-0.03	0.211
rs483082	19	45416178	*APOC1*	– –	0.01	0.11	1.8e-07	-0.04	9.2e-08	0.02	0.115
rs12721051	19	45422160	*APOC1*	– –	0.05	-0.10	3.0e-12	0.06	1.5e-12	-0.03	0.081
rs56131196	19	45422846	*APOC1*	LDL Cholesterol	0.11	-0.13	4.2e-12	0.06	2.0e-12	-0.03	0.088
rs4420638	19	45422946	*APOC1*	LDL Cholesterol	0.03	-0.10	4.2e-12	0.06	2.0e-12	-0.03	0.088
rs141622900	19	45426792	*APOC1*	LDL Cholesterol	-0.12	0.13	2.8e-38	-0.16	1.3e-36	0.09	2.76e-04
rs7259004	19	45432557	*APOC1P1*	– –	-0.02	0.10	1.6e-11	-0.07	3.6e-12	0.02	0.234
rs12721109	19	45447221	*APOC4*	LDL Cholesterol	-0.17	-0.11	1.0e-05	-0.11	2.5e-06	0.04	0.357

Supplementary Table 3: Brief overview of the PRS-Dis and PRS-PGx methods.

Disease PRS			
**Methods**	**Method Details**	**Descriptions**	**Features**
PRS-Dis-Unadj	Unadjusted	Simply add all together	Simple; may add too much noises
PRS-Dis-CT	C+T[5]	Clumping and p-value thresholding	Simple; may discard informative SNPs
PRS-Dis-LDpred2	LDpred2[1]	Bayesian regression	Most advanced disease PRS method
**PGx PRS**			
**Methods**	**Method Details**	**Descriptions**	**Features**
PRS-PGx-Unadj	Unadjusted	Simply add all together	Simple; may add too much noises
PRS-PGx-CT	C+T	Clumping and p-value (2-df test) thresholding	Simple; may discard informative SNPs
PRS-PGx-L	Lasso	Penalized regression	Assuming the prognostic and predictive effects are independent
PRS-PGx-GL	Group Lasso	Penalized regression	Assuming sparsity across groups
PRS-PGx-SGL	Sparse Group Lasso	Penalized regression	Assuming sparsity at both group and individual feature levels
PRS-PGx-Bayes	Bayesian regression	Shrink effect sizes by global-local scaling parameters	Flexibly handle various relationships between prognostic and predictive effects

Supplementary References

1. Privé, F., Arbel, J. & Vilhjálmsson, B. J. LDpred2: better, faster, stronger. *Bioinformatics* **36,** 5424–5431 (2020).

2. Vilhjálmsson, B. J. *et al.* Modeling linkage disequilibrium increases accuracy of polygenic risk scores. *Am. J. Hum. Genet.* **97,** 576–592 (2015).

3. VanLiere, J. M. & Rosenberg, N. A. Mathematical properties of the r2 measure of linkage disequilibrium. *Theoretical population biology* **74,** 130–137 (2008).

4. Ding, Y., Li, Y. G., Liu, Y., Ruberg, S. J. & Hsu, J. C. Confident inference for SNP effects on treatment efficacy. *The Ann. Appl. Stat.* **12,** 1727–1748 (2018).

5. Euesden, J., Lewis, C. M. & O'Reilly, P. F. PRSice: polygenic risk score software. *Bioinformatics* **31,** 1466–1468 (2015).
