# Supplementary material for: Pharmacogenomics polygenic risk score for drug response prediction using PRS-PGx methods
Source: Nat Commun. 2022 Sep 8;13:5278. doi: 10.1038/s41467-022-32407-9 (PMC9458667; doi:10.1038/s41467-022-32407-9)
Supplement: Supplementary file 3 — Reporting Summary [file 41467_2022_32407_MOESM3_ESM.pdf]

nature portfolio

Corresponding author(s): Judong Shen

Last updated by author(s): 07/19/2022

Reporting Summary

Nature Portfolio wishes to improve the reproducibility of the work that we publish. This form provides structure for consistency and transparency in reporting. For further information on Nature Portfolio policies, see our Editorial Policies and the Editorial Policy Checklist.

Statistics

For all statistical analyses, confirm that the following items are present in the figure legend, table legend, main text, or Methods section.

n/a	Confirmed	
☐	☒	The exact sample size (n) for each experimental group/condition, given as a discrete number and unit of measurement
☐	☒	A statement on whether measurements were taken from distinct samples or whether the same sample was measured repeatedly
☐	☒	The statistical test(s) used AND whether they are one- or two-sided<br>*Only common tests should be described solely by name; describe more complex techniques in the Methods section.*
☐	☒	A description of all covariates tested
☐	☒	A description of any assumptions or corrections, such as tests of normality and adjustment for multiple comparisons
☐	☒	A full description of the statistical parameters including central tendency (e.g. means) or other basic estimates (e.g. regression coefficient) AND variation (e.g. standard deviation) or associated estimates of uncertainty (e.g. confidence intervals)
☐	☒	For null hypothesis testing, the test statistic (e.g. F, t, r) with confidence intervals, effect sizes, degrees of freedom and P value noted<br>*Give P values as exact values whenever suitable.*
☐	☒	For Bayesian analysis, information on the choice of priors and Markov chain Monte Carlo settings
☒	☐	For hierarchical and complex designs, identification of the appropriate level for tests and full reporting of outcomes
☒	☐	Estimates of effect sizes (e.g. Cohen's d, Pearson's r), indicating how they were calculated

Our web collection on statistics for biologists contains articles on many of the points above.

Software and code

Policy information about availability of computer code

Data collection	The main focus of this paper is to propose statistical methods, with applications to existing data. Therefore, no commercial, open source and customized code or software were used to collect the data in this study.
Data analysis	The PRSPGx R package (version 0.3.0) is available at the Comprehensive R Archive Network (CRAN): https://cran.r-project.org/web/packages/PRSPGx. Other software programs used in the data analyses are: glmnet R package v4.1.1; gglasso R package v1.5; SGL R package v1.3; bigsnpr R package v1.8.1. Open Targets: https://genetics.opentargets.org; LDL-C Global Lipids Genetics Consortium GWAS summary statistics data: http://csg.sph.umich.edu/willer/public/lipids2017; 1000 Genomes Phase3 v5 reference data: http://csg.sph.umich.edu/abecasis/mach/download/1000G.Phase3.v5; LD blocks in human population: https://bitbucket.org/nygcresearch/ldetect-data.

For manuscripts utilizing custom algorithms or software that are central to the research but not yet described in published literature, software must be made available to editors and reviewers. We strongly encourage code deposition in a community repository (e.g. GitHub). See the Nature Portfolio guidelines for submitting code & software for further information.

Data

Policy information about availability of data

All manuscripts must include a data availability statement. This statement should provide the following information, where applicable:

- Accession codes, unique identifiers, or web links for publicly available datasets
- A description of any restrictions on data availability
- For clinical datasets or third party data, please ensure that the statement adheres to our policy

The LDL GWAS summary statistics data used in case study were from Global Lipids Genetics Consortium, directly downloaded from: http://csg.sph.umich.edu/willer/public/lipids2017. The 1000 Genomes Phase3 v5 reference data were downloaded from http://csg.sph.umich.edu/abecasis/mach/download/1000G.Phase3.v5. The

linkage disequilibrium (LD) blocks in human population were downloaded from: https://bitbucket.org/nygcresearch/ldetect-data. MSD's data sharing policy, including restrictions, is available at http://engagezone.msd.com/ds_documentation.php. Requests for access to the PGx GWAS summary statistics results from this IMPROVE-IT clinical study data can be submitted through the EngageZone site or via email to dataaccess@merck.com.

Field-specific reporting

Please select the one below that is the best fit for your research. If you are not sure, read the appropriate sections before making your selection.

☒ Life sciences ☐ Behavioural & social sciences ☐ Ecological, evolutionary & environmental sciences

For a reference copy of the document with all sections, see nature.com/documents/nr-reporting-summary-flat.pdf

Life sciences study design

All studies must disclose on these points even when the disclosure is negative.

Sample size

This is a methodology-based research. We developed a series of PRS-PGx (Polygenic Risk Score-Pharmacogenomics) methods, including a novel Bayesian regression approach (PRS-PGx-Bayes) for drug response prediction in pharmacogenomics GWAS. In order to demonstrate the methods' performance in the simulation and real data analysis, we used the largest datasets available. More specifically, we used a PGx GWAS data from the IMPROVE-IT trial for real data analysis to validate the results from our simulations. The sample size for the analysis (N = 5,661) was determined by the original genotype data, data quality control and a filtering step.

Briefly, 7,971 patients who provided appropriate consents for the genetic studies and had DNA with sufficient quality were genotyped using a custom Axiom™ array (Thermo Fisher Scientific, CA, USA). Genotyping was performed by BioProcessing Solutions Alliance (Piscataway, NJ, USA), and the raw data was processed following the vendor recommended guidelines. Genetic quality control (QC) steps were performed in 6,765 self-reported European subjects. Variants with missing call rate greater than 3%, as well as non-autosomal variants were excluded. Following variant QC, individuals were excluded if they had low call rates (missingness greater than 5%), gender mismatches, heterozygosity greater than 3 standard deviations from the mean of the Caucasian population, or identical and first/second degree relatives using identity-by-descent (IBD > 0.1875). Principal component analysis (PCA) was performed in a subset of variants pruned for linkage disequilibrium using EIGENSTRAT smartpca (Patterson et al., 2006), together with 1000 Genomes phase III data (1000G Consortium, 2015) as the reference. Individuals who deviated +/- 6 standard deviation from the means of 1000 Genomes European super population for principal components (PC) 1-3 were also removed. A second PCA was then performed with European-only individuals to calculate the eigenvalues to be used as covariates in the genetic analyses to correct for within population stratification. In total, 6,502 individuals of European descent, and 644,570 directly genotyped variants have been used for imputation. Imputation was performed using IMPUTE2 (Howie et al., 2009) and 1000 Genomes European panel as the reference. 9,407,967 variants with minor allele frequency (MAF) greater than 0.01, and imputation quality score (r2) greater than 0.3 were used for the subsequent genome-wide association studies (GWAS) analyses.

The subjects were further filtered by excluding subjects who had cardiovascular event prior to month 1 since cardiovascular event affected LDL-C that may not be treatment related. A total of 5,661 European subjects were included for the drug response PRS analyses.

Data exclusions

See the above "Sample size" box for the data exclusion details due to genotype data quality control (QC) and a filtering step. This genotype data QC was conducted using our Merck internal standard GWAS data QC pipeline to make sure the data for analyses were in high quality.

Replication

We do not use any independent replicates in our study. The only data we used were the IMPROVE-IT GWAS data. In our analyses, we further used 5-fold cross-validation to verify the reproducibility of the findings by using the IMPROVE-IT GWAS data. The performance from this 5-fold cross-validation procedure was successfully obtained and further summarized for the evaluation of the proposed methods.

Randomization

Patients received standard medical and interventional treatment for acute coronary syndrome and were randomly assigned, in a 1:1 ratio and in a double-blind fashion, to receive, once daily, either simvastatin (at a dose of 40 mg) plus ezetimibe (at a dose of 10 mg) (simvastatin–ezetimibe group) or simvastatin (at a dose of 40 mg) plus placebo (simvastatin-monotherapy group). Randomization was stratified according to prior use of lipid-lowering therapy, type of acute coronary syndrome, and status with respect to enrollment in the concurrent Early Glycoprotein IIb/IIIa Inhibition in Non–ST-Segment Elevation Acute Coronary Syndrome (EARLY ACS) trial.

Patients had follow-up visits at 30 days, at 4 months, and every 4 months thereafter. Patients who discontinued the study drug during the trial were generally followed by means of telephone calls. Blood samples were obtained at randomization, at 1, 4, 8, and 12 months, and yearly thereafter for those attending clinic visits.

For patients in either study group who had LDL cholesterol levels higher than 79 mg per deciliter (2.0 mmol per liter) on two consecutive measurements, the simvastatin dose was increased to 80 mg in a double-blind manner. In June 2011, in accordance with Food and Drug Administration guidance for limiting new prescriptions of 80 mg of simvastatin, patients were no longer eligible for an increased dose of simvastatin to 80 mg, and any patient who had been receiving the 80-mg dose for less than 1 year had the dose reduced to 40 mg. If an LDL cholesterol measurement on the new regimen was confirmed to be higher than 100 mg per deciliter, the study drug could be discontinued and more potent therapy initiated. The study continued until each patient had been followed for a minimum of 2.5 years and until the target number of events (5250) was reached. Five amendments to the protocol were implemented during the course of the study, including an increase in the sample size.

Blinding

The investigators were blinded to the group allocation during data collection and analysis.

Reporting for specific materials, systems and methods

We require information from authors about some types of materials, experimental systems and methods used in many studies. Here, indicate whether each material, system or method listed is relevant to your study. If you are not sure if a list item applies to your research, read the appropriate section before selecting a response.

Materials & experimental systems

n/a	Involved in the study
☒	☐ Antibodies
☒	☐ Eukaryotic cell lines
☒	☐ Palaeontology and archaeology
☒	☐ Animals and other organisms
☐	☒ Human research participants
☐	☒ Clinical data
☒	☐ Dual use research of concern

Methods

n/a	Involved in the study
☒	☐ ChIP-seq
☒	☐ Flow cytometry
☒	☐ MRI-based neuroimaging

Human research participants

Policy information about studies involving human research participants

Population characteristics

IMPROVE-IT is a multi-center, triple-blind (investigators, participants, and event adjudicators blinded to randomization) randomized phase 3b study to establish efficacy and safety of ezetimibe + simvastatin in comparison to simvastatin monotherapy in 18,144 high-risk patients with acute coronary syndrome (ACS) age 50 and older. 75.9% and 75.5% males are in Simvastatin Monotherapy and Simvastatin-Ezetimibe arms, respectively. All subjects entering the study were randomized in 1:1 ratio to either ezetimibe treatment (ezetimibe 10mg / simvastatin 40 mg combination) or placebo (simvastatin 40 mg once daily). Median follow-up in the trial was 6 years. For more details, please refer to the publication from the IMPROVE-IT clinical trial study: Cannon, C. P. et al. Ezetimibe added to statin therapy after acute coronary syndromes. N. Engl. J. Med. 372, 2387–2397 (2015) or https://www.nejm.org/doi/full/10.1056/nejmoa1410489. After genotyping and GWAS quality control and filtering steps, a total of 5,661 European subjects' genotypes, their imputed genotypes and clinical data were included for analysis of the LDL-C endpoint.

Recruitment

Please refer to the publication from the IMPROVE-IT clinical trial study: Cannon, C. P. et al. Ezetimibe added to statin therapy after acute coronary syndromes. N. Engl. J. Med. 372, 2387–2397 (2015) or https://www.nejm.org/doi/full/10.1056/nejmoa1410489 for details. In summary, men and women who were at least 50 years of age were eligible for inclusion if they had been hospitalized within the preceding 10 days for an acute coronary syndrome (an acute myocardial infarction, with or without ST-segment elevation on electrocardiography, or high-risk unstable angina). Patients were required to have an LDL cholesterol level of 50 mg per deciliter (1.3 mmol per liter) or higher. For participants who were not receiving long-term lipid-lowering therapy, the maximum LDL cholesterol level for enrollment was 125 mg per deciliter (3.2 mmol per liter); for participants who were receiving lipid-lowering therapy, the maximum level was 100 mg per deciliter (2.6 mmol per liter). The LDL cholesterol level for eligibility was measured locally within the first 24 hours after onset of the acute coronary syndrome. Key exclusion criteria were planned coronary-artery bypass grafting for the acute coronary syndrome event, creatinine clearance of less than 30 ml per minute, active liver disease, or use of statin therapy that had LDL cholesterol–lowering potency greater than 40 mg of simvastatin.

Ethics oversight

The ethics committee at each participating center approved the protocol and amendments. The IMPROVE-IT trial was carried out in accordance with the Declaration of Helsinki, current guidelines on Good Clinical Practices and local ethical and legal requirements. There are representatives from the 1158 enrolling centers in 39 participating countries in the IMPROVE-IT trial. It is not convenient to list all of them in this box. Please refer to the publication from the IMPROVE-IT clinical study: Cannon, C. P. et al. Ezetimibe added to statin therapy after acute coronary syndromes. N. Engl. J. Med. 372, 2387–2397 (2015) or https://www.nejm.org/doi/full/10.1056/nejmoa1410489 for more details. More specifically, the full names of the centers can be found at https://www.nejm.org/doi/suppl/10.1056/NEJMoa1410489/suppl_file/nejmoa1410489_appendix.pdf.

Note that full information on the approval of the study protocol must also be provided in the manuscript.

Clinical data

Policy information about clinical studies

All manuscripts should comply with the ICMJE guidelines for publication of clinical research and a completed CONSORT checklist must be included with all submissions.

Clinical trial registration

https://clinicaltrials.gov/ct2/show/NCT00202878

Study protocol

The IMPROVE-IT study protocol is at https://www.nejm.org/doi/suppl/10.1056/NEJMoa1410489/suppl_file/nejmoa1410489_protocol.pdf. Please refer to the publication from the IMPROVE-IT clinical trial study: Cannon, C. P. et al. Ezetimibe added to statin therapy after acute coronary syndromes. N. Engl. J. Med. 372, 2387–2397 (2015) or https://www.nejm.org/doi/full/10.1056/nejmoa1410489 for more details.

Patients received standard medical and interventional treatment for acute coronary syndrome and were randomly assigned, in a 1:1 ratio and in a double-blind fashion, to receive, once daily, either simvastatin (at a dose of 40 mg) plus ezetimibe (at a dose of 10 mg) (simvastatin–ezetimibe group) or simvastatin (at a dose of 40 mg) plus placebo (simvastatin-monotherapy group). Randomization was stratified according to prior use of lipid-lowering therapy, type of acute coronary syndrome, and status with respect to enrollment in the concurrent Early Glycoprotein IIb/IIIa Inhibition in Non–ST-Segment Elevation Acute Coronary Syndrome (EARLY ACS) trial.

Patients had follow-up visits at 30 days, at 4 months, and every 4 months thereafter. Patients who discontinued the study drug during the trial were generally followed by means of telephone calls. Blood samples were obtained at randomization, at 1, 4, 8, and 12 months, and yearly thereafter for those attending clinic visits.

For patients in either study group who had LDL cholesterol levels higher than 79 mg per deciliter (2.0 mmol per liter) on two consecutive measurements, the simvastatin dose was increased to 80 mg in a double-blind manner. In June 2011, in accordance with Food and Drug Administration guidance for limiting new prescriptions of 80 mg of simvastatin, patients were no longer eligible for an increased dose of simvastatin to 80 mg, and any patient who had been receiving the 80-mg dose for less than 1 year had the dose reduced to 40 mg. If an LDL cholesterol measurement on the new regimen was confirmed to be higher than 100 mg per deciliter, the study drug could be discontinued and more potent therapy initiated. The study continued until each patient had been followed for a minimum of 2.5 years and until the target number of events (5250) was reached. Five amendments to the protocol were implemented during the course of the study, including an increase in the sample size.

Data collection

There are representatives from the 1158 enrolling centers in 39 participating countries in the IMPROVE-IT trial. It is not convenient to list all of them in this box. Please refer to the publication from the IMPROVE-IT clinical trial study: Cannon, C. P. et al. Ezetimibe added to statin therapy after acute coronary syndromes. N. Engl. J. Med. 372, 2387–2397 (2015) or https://www.nejm.org/doi/full/10.1056/nejmoa1410489 for details. More specifically, the data collection was conducted at the centers, which can be found at https://www.nejm.org/doi/suppl/10.1056/NEJMoa1410489/suppl_file/nejmoa1410489_appendix.pdf.

Outcomes

The endpoint analyzed in our case study is the response to treatment defined as the log-fold change of low-density lipoproteins cholesterol (LDL-C) from baseline (QE) to 1 month (i.e. log(LDL-C at 1 month / LDL-C at QE)).
